# Expansion of the diversity of dispersin scaffolds

**DOI:** 10.1107/S205979832500110X

**Published:** 2025-02-28

**Authors:** Alexandra Males, Olga V. Moroz, Elena Blagova, Astrid Munch, Gustav H. Hansen, Annette H. Johansen, Lars H. Østergaard, Dorotea R. Segura, Alexander Eddenden, Anne V. Due, Martin Gudmand, Jesper Salomon, Sebastian R. Sørensen, João Paulo L. Franco Cairo, Mark Nitz, Roland A. Pache, Rebecca M. Vejborg, Sandeep Bhosale, David J. Vocadlo, Gideon J. Davies, Keith S. Wilson

**Affiliations:** a York Structural Biology Laboratory, Department of Chemistry University of York YorkYO10 5DD United Kingdom; b Novonesis A/S, Biologiens Vej 2, 2800Kongens Lyngby, Denmark; c Department of Chemistry University of Toronto Toronto Ontario Canada; d Novonesis A/S, Krogshøjvej 36, 2880Bagsvaerd, Denmark; e Department of Chemistry Simon Fraser University Burnaby British ColumbiaV5A 1S6 Canada; University of Cambridge, United Kingdom

**Keywords:** enzyme catalysis, glycoside hydrolases, poly-*N*-acetylglucosamine, protein crystallography, GH20

## Abstract

Microorganisms are known to secrete copious amounts of extracellular polymeric substances (EPS) that form complex matrices around the cells to shield them against external stresses, to maintain structural integrity and to influence their environment. Many microorganisms also secrete enzymes that are capable of remodelling or degrading EPS in response to various environmental cues. One key enzyme class is the poly-β-1,6-linked *N*-acetyl-d-glucosamine (PNAG)-degrading glycoside hydrolases, of which the canonical member is dispersin B (DspB) from CAZy family GH20. We sought to test the hypothesis that PNAG-degrading enzymes would be present across family GH20, resulting in expansion of the sequence and structural space and thus the availability of PNAGases. Phylogenetic analysis revealed that several microorganisms contain potential DspB-like enzymes. Six of these were expressed and characterized, and four crystal structures were determined (two of which were in complex with the established GH20 inhibitor 6-acetamido-6-deoxy-castanospermine and one with a bespoke disaccharide β-1,6-linked thiazoline inhibitor). One enzyme expressed rather poorly, which restricted crystal screening and did not allow activity measurements. Using synthetic PNAG oligomers and MALDI-TOF analysis, two of the five enzymes tested showed preferential endo hydrolytic activity. Their sequences, having only 26% identity to the pioneer enzyme DspB, highlight the considerable array of previously unconsidered dispersins in nature, greatly expanding the range of potential dispersin backbones available for societal application and engineering

## Abstract 2

Five GH20-family dispersins were characterized for activity on aryl glycosides and poly-β-1,6-linked *N*-acetyl-d-glucosamine oligomers and three crystal structures were solved, including two complexes with bound inhibitors.

## 1. Introduction

In nature, microorganisms actively react to and influence the environment in which they live by producing and secreting a wide range of biological molecules and chemical compounds. This allows them to protect themselves, to compete in and to exploit their immediate surroundings. Many microbial species are known to produce copious amounts of extracellular polymeric substances (EPS), which can protect them against multiple environmental stresses (Yin *et al.*, 2019 ▸). While the exact composition of the extracellular matrix depends largely on the microbial species and the environmental cues governing expression, it usually consists of a complex mixture of molecules; this includes proteins, nucleic acids and exo­polysaccharides. Poly-*N*-acetylglucosamine (PNAG), also known as polysaccharide intracellular adhesin (PIA), is a homopolymer of linear chains of partially de-*N*-acetylated β-1,6-linked *N*-acetyl-d-glucosamine (β-1,6-GlcNAc). This key exopolysaccharide is produced by a wide range of microbial species, including both Gram-negative and Gram-positive bacteria, such as *Escherichia coli*, *Staphylococcus aureus* (Cramton *et al.*, 1999 ▸), *Actinobacillus actinomycetem­comitans* (Kaplan *et al.*, 2004 ▸) and *Bacillus subtilis* (Roux *et al.*, 2015 ▸), as well as several protozoan and fungal species (Cywes-Bentley *et al.*, 2013 ▸); these species cause over 50% of nosocomial infections (Jamal *et al.*, 2018 ▸). A collection of four proteins are involved in the biosynthesis, modification and excretion of PNAG and are encoded either by the *pgaABCD* genes identified in the Gram-negative bacteria *A. actinomycetemcomitans* (Kaplan *et al.*, 2004 ▸), *E. coli* (Wang *et al.*, 2004 ▸) and *Yersinia pestis* (Lillard *et al.*, 1997 ▸) or the *icaABCD* genes of the intracellular adhesion (*ica*) locus present in Gram-positive bacteria such as *Staphylococcus* spp. (Cramton *et al.*, 1999 ▸). Most of the PgaABCD proteins share sequence and, where known, structural homology with their *ica* operon counterparts. Some species of bacteria, fungi and protozoa have been shown to produce PNAG although they lack a known genetic locus for its synthesis (Cywes-Bentley *et al.*, 2013 ▸), indicating a convergent evolutionary mechanism for the acquisition of PNAG synthesis with potentially considerable significance for microbial biology. Due to the ubiquity of PNAG, its production has been proposed to rival that of other common polysaccharides such as chitin and cellulose (Cywes-Bentley *et al.*, 2013 ▸).

The reservoir of cells within a sessile community, whilst beneficial to pathogen survival, can be detrimental to the external environment. The colonization of bacteria on abiotic manmade and natural surfaces causes significant problems in the food sector, medicine (for example drug resistance, mammalian infections, adhesion to implants and valves) and industry (for example machine fouling) (Donlan, 2002 ▸). Application of antibiotics directly onto sessile cells requires 10–1000 times the minimum inhibitory concentration of antibiotics needed to inhibit the planktonic form (Ceri *et al.*, 1999 ▸). New strategies to reduce the impact of antibacterial resistance while treating infections are urgently required.

Several microorganisms secrete enzymes that are capable of directly degrading EPS, enabling them to control the composition of the matrix in which they are situated and facilitate dispersion under unfavourable conditions. PNAG-hydrolyzing enzymes have been classed into two separate glycoside hydrolase CAZy families, GH20 and GH153 (http://www.cazy.org; Drula *et al.*, 2022 ▸). The division was based on limited sequence similarity, the presence or absence of PNAG-biosynthesis genes located within the same operon, the presence of a second deacetylase domain and the preference for deacetylated GlcNAc in different active-site subsites.

Translated from the *pgaABCD* operon, PgaB is a dual-functioning enzyme which is a fusion of CAZy family GH153 and CE4 enzymes (Lombard *et al.*, 2014 ▸). The N-terminal domain acts as a deacetylase for PNAG, resulting in positively charged PNAG, allowing it to interact more readily with the negatively charged cell membrane surface. The C-terminal domain functions as a glycoside hydrolase and cleaves the β-1,6-glycosidic bonds of the PNAG polysaccharide, recognizing a GlcN–GlcNAc–GlcNAc motif in the −3, −2, −1 subsites (Little *et al.*, 2018 ▸). Structures of PgaB from *Bordetella bronchiseptica* and *E. coli* have provided vital insight into the catalytic mechanisms (Little *et al.*, 2018 ▸).

Dispersin B (DspB), isolated from *A. actinomycetemcomitans*, was one of the first identified examples of an enzyme that is capable of degrading PNAG (Kaplan, Meyenhofer *et al.*, 2003 ▸). The enzyme is a β-hexosaminidase belonging to CAZy glycoside hydrolase family GH20 and its crystal structure has been reported (Lombard *et al.*, 2014 ▸; Ramasubbu *et al.*, 2005 ▸). DspB is not merely the canonical dispersin; it is currently quite unique in the academic literature. Although the CAZy GH20 family contains over 10 000 members (>129 proteins have been characterized with 27 crystal structures, the first being the chitobiase from *Serratia marcescens*; Tews *et al.*, 1996 ▸), only two GH20 enzymes have been classified as a dispersin: DspB from *A. actinomycetemcomitans*, mentioned above, and DspB from *A. pleuropneumoniae* (Kaplan *et al.*, 2004 ▸). This inspired us to search the genomic resource for new dispersins.

DspB is best considered to be an endo enzyme with a kinetic preference for deacetylated substrates (that is, glucosamine, GlcN) in both the −2 and +2 subsites. This is reflected in a faster hydrolysis of partially deacetylated substrates (Wang *et al.*, 2019 ▸). This preference has been attributed to the charge–charge interactions between the cationic glucosamine and anionic aspartates: Asp147 in the +2 subsite and Asp245 in the +1 subsite (Breslawec *et al.*, 2021 ▸). However, when DspB is provided with an unfavourable substrate, it can also act in an ‘exo’ manner, bypassing deacetylated units to ensure that the *N*-acetyl group of GlcNAc lies in the −1 subsite (Wang *et al.*, 2019 ▸). The only published crystal structure of DspB (PDB entry 1yht; Ramasubbu *et al.*, 2005 ▸) contains glycerol in the −1 subsite and this structure has been used to model ligands in the active site. There are no crystal structures of dispersins in complex with a more informative ligand.

In order both to expand the sequence and structural diversity of known dispersins and to provide structural insight into ligand binding, here we report the cloning, expression and characterization, through inhibition studies and their potential to hydrolyse colourimetric and fluorometric glycoside substrates and bacterially derived PNAG, of five new bacterial GH20 dispersins, henceforth termed DispTs, DispTs2 and DispTs3 (from *Terribacillus saccharophilus*), DispLp (from *Lactiplantibacillus paraplantarum*, formerly known as *Lactobacillus paraplantarum*) and DispSf (from *Mammaliicoccus fleurettii*, formerly known as *Staphylococcus fleurettii*). A sixth sample initially included in the study, DispCo (from *Curtobacterium oceanosedimentum*, chosen to further increase the dispersin diversity; we aimed to have enzymes from separate clades), could only be produced in very limited quantities and was easily degraded, which did not allow activity experiments. To summarize, the new dispersins are phylogenetically distinct from DspB; separated by different phyla, the new dispersins are present in Actinobacteria and Firmicutes compared with Proteobacteria. The dispersins were exposed to both synthesized (fully acetylated) and crude (partially deacetylated) PNAG; hydrolysis of biologically produced PNAG was confirmed through an indirect enzyme-linked immunosorbent assay (ELISA) and hydrolysis of synthetic PNAG was observed to varying degrees of efficiency via matrix-assisted laser desorption/ionization coupled to time-of-flight mass spectrometry (MALDI-TOF-MS). Four crystal structures were determined for three of these enzymes: one structure without a ligand, one in complex with a bespoke disaccharide β-1,6-linked thiazoline inhibitor, and two in complex with the inhibitor 6-acetamido-6-deoxy-castanospermine (6-Ac-Cas) in the −1 subsite. Ongoing research into the medical and industrial applications of DspB highlights the need to enhance the stability, large-scale production and activity of DspB (Yakandawala *et al.*, 2009 ▸; Seijsing *et al.*, 2020 ▸; Tan *et al.*, 2015 ▸). With the sequences of these new dispersins varying in identity from 26% to 37%, this greatly expands the array of potential dispersin templates available for societal application and engineering.

## 2. Materials and methods

### 2.1. Phylogenetic analysis, structure predictions and comparisons

The dispersin phylogenetic tree was constructed by aligning the sequences taken from a *BLAST* search against DspB using *ClustalW* (Thompson *et al.*, 1994 ▸). The tree was constructed and visualized using *MEGA X* (Kumar *et al.*, 2018 ▸). For structure predictions (Varadi *et al.*, 2024 ▸; Jumper *et al.*, 2021 ▸) *alphafold*2_*multimer_v*3 was used, creating relaxed models using all five different *AlphaFold*2 network variations, and the best-ranked model was picked (ranking based on the predicted local distance difference test; pLDDT). Structure comparisons were carried out using *SSM* (Krissinel & Henrick, 2004 ▸), as incorporated in *Coot* (Emsley *et al.*, 2010 ▸).

### 2.2. Genetic cloning of dispersins

The genes of interest chosen for cloning and characterization are listed in Table 1 ▸ with their corresponding donor organisms and nucleotide-sequence accession numbers. The genes encoding DispTs, DispTs2, DispTs3, DispLp, DispSf and DispCo were purchased as codon-optimized synthetic genes for *B. subtilis* expression from ThermoFisher Scientific and GeneArt. The gene encoding DspB was purchased from GenScript Biotech and codon-optimized for *E. coli* expression.

The synthetic dispersin genes were inserted into a *Bacillus*expression plasmid as described previously (Moroz *et al.*, 2017 ▸). The DNA encoding the mature polypeptide, predicted by *SignalP* (Bendtsen *et al.*, 2004 ▸), was cloned with the In-Fusion HD EcoDry Cloning Kit in frame with the *B. clausii* secretion signal peptide, replacing the native secretion signal sequence, followed by a polyhistidine tag. The residue numbering of the dispersins in the sequence alignment and within the PDB files starts from the beginning of the mature peptide.

Recombinant *B. subtilis* clones containing the individual integrated expression constructs were selected and cultivated on a rotary shaking table in 500 ml baffled Erlenmeyer flasks each containing 100 ml LB medium supplemented with 34 mg l^−1^ chloramphenicol. The culture was cultivated for three days at 30°C. The enzyme-containing supernatants were harvested by centrifuging the culture broth for 30 min at 15 000*g* and the enzymes were purified as described below.

Residues 21–381 of DspB were cloned into the NdeI and KpnI restriction-enzyme cleavage sites of the pET-29b plasmid, which contains a C-terminal hexahistidine tag.

### 2.3. Fermentation, gene expression and protein purification

The culture supernatants were filtered through a Nalgene 0.2 µm filtration unit to remove the rest of the *B. subtilis* host cells. The 0.2 µm filtrates were transferred to 20 m*M* MES–NaOH pH 6.0 on a G25 Sephadex column (GE Healthcare). The transferred solutions were applied onto a Source Q column (GE Healthcare) equilibrated in 20 m*M* MES–NaOH pH 6.0. After washing the column extensively with equilibration buffer, the proteins were eluted with a linear NaCl gradient (0–1.0 *M* NaCl) over five column volumes. Fractions were collected during elution and analysed by SDS–PAGE. Fractions for which only one band was seen after Coomassie staining were pooled and used for further experiments.

The protocol used for the gene expression and protein purification of DspB is described in Ramasubbu *et al.* (2005 ▸).

### 2.4. Enzymatic assays using 4-nitrophenyl-β-*N*-acetyl-d-glucosaminide (*p*NP-GlcNAc)

Due to the problems with expression and purification resulting in a limited quality and quantity of the DispCo sample, it was excluded from all activity experiments. A sample of each of the other five dispersins was taken after cell growth, purified and stored in 50 m*M* HEPES, 100 m*M* NaCl pH 7. The purified enzyme was subsequently normalized to 25 µ*M* in MQ/0.01% Triton X-100 and further diluted in buffer (100 m*M* acetic acid, 100 m*M* MES, 100 m*M* HEPES, 100 m*M* glycine pH 5) to a final assay concentration of 1500, 300 or 60 n*M*. The dispersin was reacted with 6 m*Mp*NP-GlcNAc (CAS No. 459-18-5) for 30 min under gentle shaking (300 rev min^−1^). The total volume of the reaction solution was 100 µl. Reaction was stopped by the addition of 100 µl 0.6 *M* Na_2_HCO_3_ pH 10.3. After allowing the pH to equilibrate for 20 min under gentle shaking (150 rev min^−1^), the endpoint absorbance was measured at 405 nm. All data points were blank-corrected using a sample with 0 n*M* dispersin.

### 2.5. Enzymatic assays using 4-methylumbelliferyl-*N*-acetyl-β-d-glucosaminide (4-MU-GlcNAc)

A sample of each dispersin was taken after cell growth, purified and stored in 50 m*M* HEPES, 100 m*M* NaCl pH 7 or similar. The purified enzyme was subsequently normalized to 20 n*M* in MQ/0.01% Triton X-100 and further diluted in buffer (20 m*M* acetic acid, 20 m*M* MES, 20 m*M* HEPES, 20 m*M* glycine pH 5) to a final assay concentration of 20, 4 or 0.8 n*M*. The dispersin was reacted with 5 m*M* 4-MU-GlcNAc (CAS No. 37067-30-4) for 20 min under gentle shaking (150 rev min^−1^). The total volume of the reaction solution was 100 µl. Reaction was stopped by the addition of 100 µl 0.6 *M* Na_2_HCO_3_ pH 10.3. After allowing the pH to equilibrate for 5 min under gentle shaking (150 rev min^−1^), the endpoint fluorescence was measured using excitation at 368 nm and emission at 448 nm. All data points were blank-corrected using a sample with 0 n*M* dispersin.

### 2.6. Enzyme hydrolysis of synthetic PNAG

Synthetic PNAG was produced by an acid-reversion reaction with HF–pyridine as described previously (Leung *et al.*, 2009 ▸). The mixed oligomers were fractionated on a BioGel P4 column in dH_2_O and a fraction with lengths of between 6 and 10 GlcNAc units was used in the assays.

The enzymes and PNAG were diluted into 20 m*M* ammonium acetate pH 6.0 to final concentrations of 10 µ*M* and 1 mg ml^−1^, respectively. In a shaking block, 10 µl reactions were conducted in triplicate at 37°C for 20 h. A 1 µl sample was loaded onto a MALDI 384 ground-steel target plate TF (Bruker Daltonics) and mixed on-plate with 10 mg ml^−1^ 2,5-dihydroxybenzoic acid (DHB) dissolved in 50% aceto­nitrile, 0.1% trifluoroacetic acid followed by air-drying to allow crystallization. The data were collected using an ultrafleXtreme (MALDI-TOF/TOF, Bruker), with the smartbeam-II laser set to 2 kHz in positive-ion mode. The laser power was set to 60% and the ions were acquired in reflector mode (mass range 0–3000 Da) for MS analysis. The data were processed in the Bruker *flexAnalysis* software using a red phosphorus standard as a calibrant. A control reaction with the substrate and BSA was conducted, and no hydrolysis products were observed. Furthermore, the five enzymes were incubated with a chitin heptasaccharide and this substrate was partially degraded.

### 2.7. Extraction of PNAG from *Pseudomonas fluorescens*

A crude PNAG extract was prepared from *P. fluorescens*as follows. The strain was grown in M63 [15 m*M* (NH_4_)_2_SO_4_, 100 m*M* KH_2_PO_4_, 1.8 µ*M* FeSO_4_, 1 m*M* MgSO_4_·7H_2_O, 0.4%(*w*/*v*) glycerol, 0.2%(*w*/*v*) casamino acids, 0.0001%(*w*/*v*) thiamine] in Corning CellBIND 225 cm^2^ angled neck cell-culture flasks with a vent cap (400 ml per flask) at 20°C for three days under static conditions. After cultivation, the culture was pelleted by centrifugation (10 min, 8000*g*, 25°C), and resuspended in 3 *M* NaCl to extract the surface-associated EPS much as described previously (Chiba *et al.*, 2015 ▸). The PNAG-containing supernatant obtained after a subsequent centrifugation step (10 min, 5000*g*, 25°C) was stored at −20°C until use.

### 2.8. Quantification of PNAG by indirect ELISA

The crude PNAG extract was diluted 1:10 in 1× PBS and subjected to enzymatic treatment for 1 h at 37°C prior to the ELISA. Nontreated samples and samples treated with heat-inactivated dispersin B were used as controls (1 h, 100°C). For quantification of the residual PNAG, ELISA plates (Nunc MultiSorp) were coated with the PNAG samples for 1 h at room temperature, rinsed and blocked overnight at 4°C (in PBST + 1% BSA). The primary antibody solution [human Anti-PNAG antibody (TAB-799CL), Creative Biolabs, diluted 1:5000 in PBST + 1% BSA] was added and the plates were incubated for 1 h at room temperature. The plates were then rinsed in PBST and treated with HRP-conjugated anti-human secondary antibodies (goat anti-human, Sigma–Aldrich, diluted 1:5000 in PBST) for 1 h, followed by development using the TMB plus2 ready-to-use 3,3′,5,5′-tetramethyl­benzidine-based chromogenic solution according to the manufacturer’s instructions (KemEnTec Dianostics). The absorbance was measured at 450 nm using a spectrophoto­meter.

### 2.9. Dissociation-constant measurement by isothermal titration calorimetry (ITC)

ITC was performed for three of the enzymes. 200 µ*M* DispTs2 or DispLp and 2000 µ*M* GlcNAc-castanospermine were buffer-matched into 20 m*M* HEPES pH 7.5 or pH 7.0 and 50 m*M* NaCl. 50 µ*M* DispSf and 500 µ*M* GlcNAc-castanospermine were buffer-matched into 50 m*M* HEPES pH 7.5. ITC was performed using a MicroCal ITC200 calorimeter, where GlcNAc-castanospermine was added by syringe with 20 injections to the protein solution in the calorimeter cell at 25°C. A control used GlcNAc-castanospermine injected into buffer in the cell. The dissociation constant (*K*_d_), number of sites (*N*) and enthalpy change (Δ*H*) were calculated using one-site fitting within the *MicroCal PEAQ-ITC Analysis* software (Malvern Panalytical) after subtraction of the control.

### 2.10. Crystallization of the dispersins

Initial crystallization screening was carried out for all six enzymes, including DispCo, using sitting-drop vapour diffusion with drops set up using a Mosquito Crystal liquid-handling robot (STP LabTech) with 150 nl protein solution plus 150 nl reservoir solution in 96-well format plates (MRC 2-well crystallization microplates, SWISSCI) equilibrated against 54 µl reservoir solution. The initial experiments were carried out at room temperature with a variety of commercial screens. We obtained crystals for three of the samples during screening. DispTs was dropped because of its high sequence similarity to DispTs3, DispCo had an additional N-terminal domain, most probably connected to the catalytic domain by a flexible linker, and was prone to rapid degradation, with both factors negatively influencing crystallization, and DispSf did not lead to diffraction-quality crystals.

To crystallize DispTs3 (13.3 mg ml^−1^), an initial seeding stock was made from crystals from JCSG condition H7: 0.2 *M* ammonium sulfate, bis-Tris pH 5.5, 25% PEG 3350. The final crystals were obtained after several rounds of microseed matrix screening (MMS; D’Arcy *et al.*, 2014 ▸; Shaw Stewart *et al.*, 2011 ▸; Shah *et al.*, 2005 ▸) using an Oryx8 robot (Douglas Instruments) into Morpheus condition B3: 0.09 *M* Halogens Mix, 30% Glycerol/PEG 4000 Mix.

Crystals of DispTs2 (36 mg ml^−1^) were obtained using MMS (with DispTs3 seeding stocks and then stocks from the new hits for DispTs2) into MPD Screen condition E7: 0.1 *M* citric acid pH 4.0, 20% MPD. The crystals were co-crystallized with 5 m*M* 6-Ac-Cas (PDB ligand code GC2).

A seeding stock made from crystals of DispLp (19 mg ml^−1^) in Hampton Research Crystal Screen condition D10 (0.2 *M* calcium acetate, 0.1 *M* sodium cacodylate pH 6.5, 18% PEG 8000) was added by MMS into the PACT Screen (Molecular Dimensions). Crystals were obtained in PACT condition A10: 0.2 *M* calcium chloride, 0.1 *M* sodium acetate pH 5.0, 20% PEG 6000). The crystals were co-crystallized with 5 m*M* 6-Ac-Cas (PDB ligand code GC2) and cryoprotected using ∼30% ethylene glycol.

### 2.11. Data collection, structure solution and refinement

All computation was carried out using programs from the *CCP*4 suite (Agirre *et al.*, 2023 ▸). Data were collected at Diamond Light Source (DLS) and processed with *xia*2 (Winter, 2010 ▸). The data-collection and processing statistics are given in Table 2 ▸. The structures of DispTs3 and DispLp were solved by molecular replacement using *MOLREP* (Vagin & Teplyakov, 2010 ▸) with PDB entry 1yht (dispersin B from *A. actinomycetemcomitans*) as the search model, which was selected using *MrBUMP*–*CCP*4*mg* to provide a sculpted model (Ramasubbu *et al.*, 2005 ▸; Keegan & Winn, 2007 ▸). The structure of DispTs2 was solved by molecular replacement using *MOLREP* with DispTs3 as the model (Vagin & Teplyakov, 2010 ▸). The chains in all of the protein structures were traced using *Buccaneer* and the structures were refined with *REFMAC* iterated with manual model correction using *Coot* (Murshudov *et al.*, 2011 ▸; Emsley *et al.*, 2010 ▸; Cowtan, 2006 ▸). The quality of the final models was validated using *MolProbity* as part of the *Phenix* package (Adams *et al.*, 2011 ▸; Chen *et al.*, 2010 ▸).

## 3. Results

### 3.1. Phylogenetic analysis unveils new dispersin scaffolds

To date, more than 300 bacterial species have been shown to produce PNAG/PIA (Cywes-Bentley *et al.*, 2013 ▸) along two distinct evolutionary trajectories involving either the *pga* or *ica* machinery (Bundalovic-Torma *et al.*, 2020 ▸). Notably, the mechanisms for cell detachment by sloughing through external forces, proteases or nucleases and detergents, for example phenol-soluble modulins, are dependent on the composition of the extracellular matrix (Guilhen *et al.*, 2017 ▸). DspB is to date the sole characterized GH20 dispersin subfamily member, suggesting that there is probably a plethora of unidentified dispersin or dispersin-like enzymes. This led us to search for a correlation between bacterial species that contain genes encoding putative dispersin enzymes and the presence of a PNAG operon. A *BLAST* search using the sequence for DspB retrieved over 1000 related sequence results with an *E*-value below 10^−20^.

**Figure 1:**
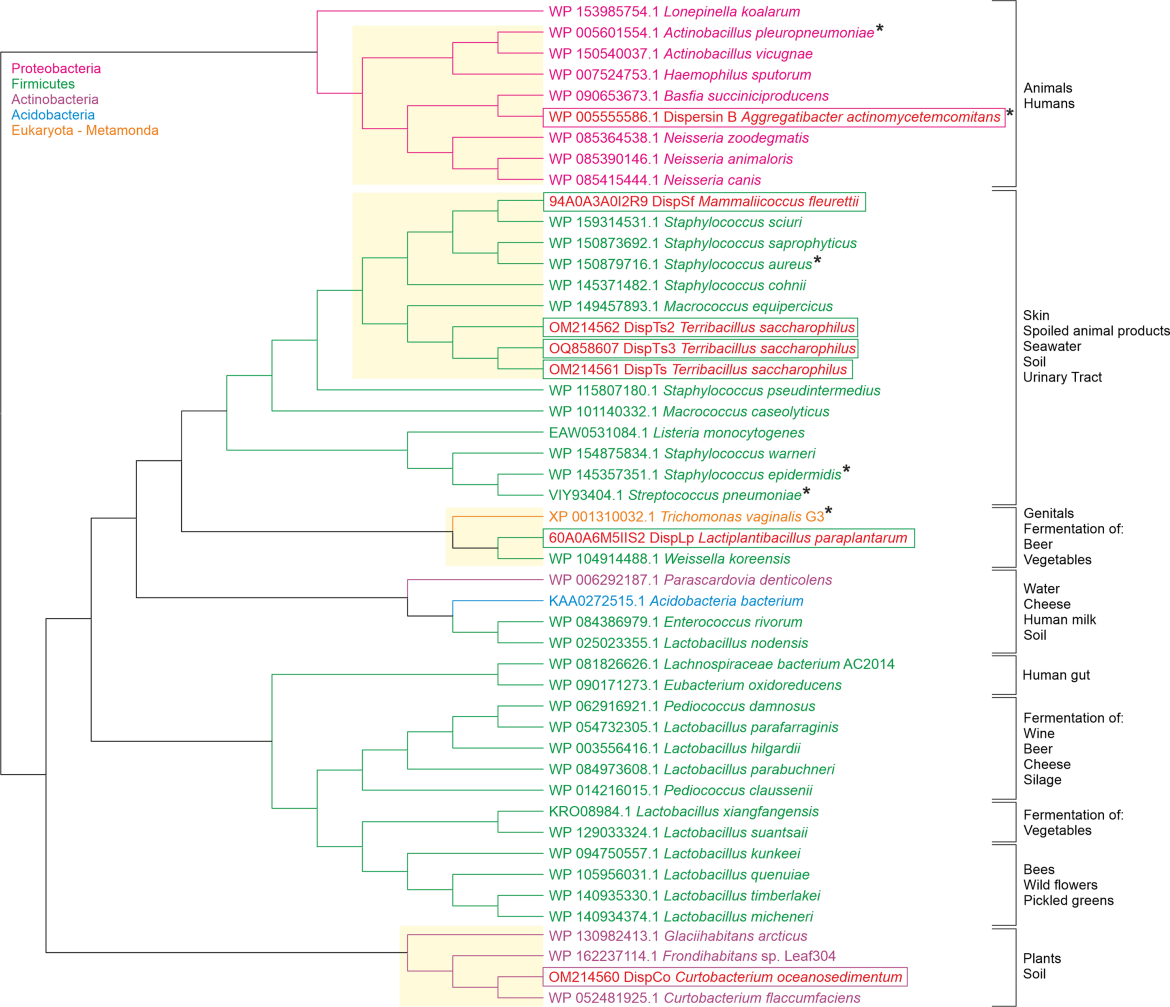
Distinct phylogenetic clades separate the dispersins. A cladogram describing the distribution of predicted GH20 dispersin members across different phyla of Bacteria and Eukaryota. The dispersins discussed in the paper are identified in the boxes coloured according to their phylum. Asterisks indicate species for which there is evidence of PNAG expression (Cramton *et al.*, 1999 ▸; Kaplan, Ragunath *et al.*, 2003 ▸; Cywes-Bentley *et al.*, 2013 ▸; Izano *et al.*, 2007 ▸; Mack *et al.*, 1996 ▸). Clades containing the new dispersin enzymes are highlighted with a yellow background.

Sequence alignment of 50 ‘dispersin’ enzymes from different bacterial species allowed the construction of a cladogram using maximum-likelihood methods (Fig. 1 ▸). Three distinct clades could be distinguished in the phyla Proteobacteria, Actinobacteria and Firmicutes. The Firmicute clade also contained members of the Acidobacteria and Actinobacteria phyla and *Trichomonas vaginalis* from the Eukaryota Metamonada phylum. Similarities between enzyme sequence, clade and the location of bacterial isolation were evident, for example the distinct clade of Actinobacteria can be isolated from plants and soil. No evolutionary information can be deduced from the cladogram since the outgroup, containing the phylum that evolved first, could not be resolved.

From these 50 sequences, six enzymes, representing three different clades, were selected for further analysis. Those chosen are crucial for obtaining dispersin diversity phylogenetically distinct from the clade containing the well characterized dispersin B from *A. actinomycetemcomitans* (Fig. 1 ▸). A clade containing DispLp, which has 30% sequence identity to DspB along with potential dispersins from two other species, is separate from members of a second clade containing DispSf (26% sequence identity to DspB), DispTs3 (26% sequence identity to DspB) and DispTs and DispTs2 (26% and 28% sequence identity to DspB, respectively). A third clade was identified containing the Actinobacterium phylum and DispCo (36% identity to DspB).

### 3.2. The putative dispersins were active against aryl glycosides

**Figure 2:**
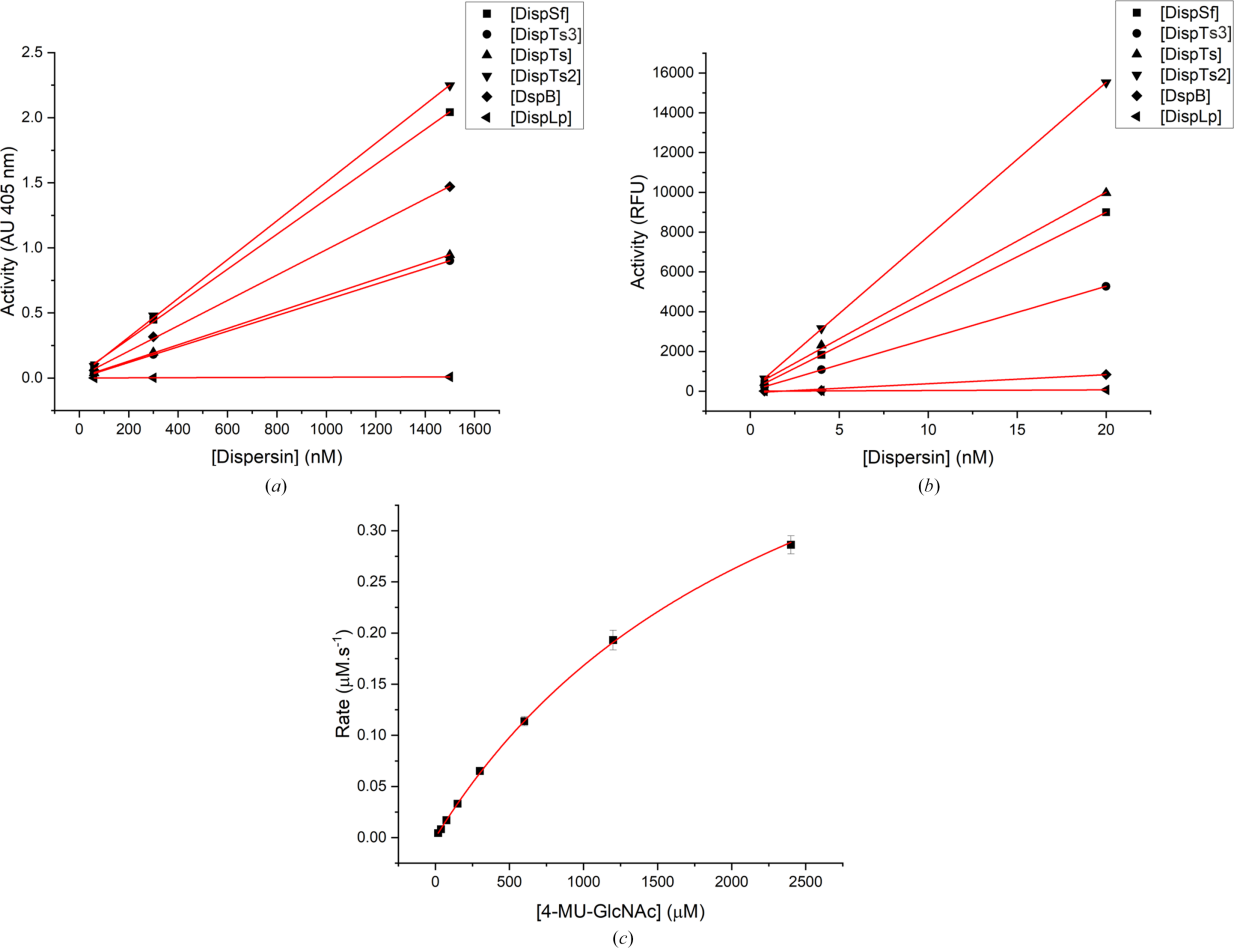
Dispersin activity–concentration profiles for two different substrates. All profiles shown in (*a*) and (*b*) are linear under the given conditions. (*a*) Activity–concentration profiles of all tested dispersins against *p*NP-GlcNAc substrate. (*b*) As (*a*) but against 4-MU-GlcNAc substrate. (*c*) Michaelis–Menten kinetics of DispTs2 using 4-MU-GlcNAc as the substrate.

To determine the activity of the putative dispersins, we first performed activity–concentration kinetics with two different aryl glycosides: *p*NP-GlcNAc and 4-MU-GlcNAc. The activity–concentration profiles of the two assays showed a linear relationship for all dispersin enzymes measured under the chosen conditions (Figs. 2 ▸*a* and 2 ▸*b*). DispLp showed very low activity against both substrates, while DispTs2 showed the highest activity of the tested dispersins. Although DispTs2 and DispTs3 have the highest sequence similarity, DispTs2 was more active on both aryl glycosides. There were slight differences in substrate specificity when directly comparing the two substrates, most notably for DspB (which showed a preference for *p*NP-GlcNAc) but also DispSf (preference for *p*NP-GlcNAc) and DispTs (preference for 4-MU-GlcNAc). DispLp has a high sequence identity of 30% to DspB, but in marked contrast showed very little activity on these substrates.

Since the highest measured activity was the hydrolysis of 4-MU-GlcNAc by DispTs2, determination of the Michaelis–Menten kinetic parameters was attempted. Although saturating concentrations of 4-MU-GlcNAc were not possible, an observed *K*_m_ of 2.5 ± 0.1 m*M* and *V*_max_ of 0.59 ± 0.02 µ*M* s^−1^ were estimated (Fig. 2 ▸*c*). Although the enzymes are active on these substrates, it is likely that neither 4-MU nor *p*NP were well tolerated in the active site of the enzymes, or the substrates need to have longer oligosaccharide moieties consistent with extended subsites and an endo activity of the dispersins (Wang *et al.*, 2020 ▸).

### 3.3. The dispersins show activity against fully acetylated and partially deacetylated PNAG

After observing the activity of the putative dispersins against aryl glycosides, five enzymes (excluding DispCo) were tested for activity on PNAG. The hydrolysis was monitored using two techniques: MALDI-TOF-MS on fully synthetic (and fully acetylated) PNAG and an indirect ELISA on PNAG isolated from *P. fluorescens*.

**Figure 3:**
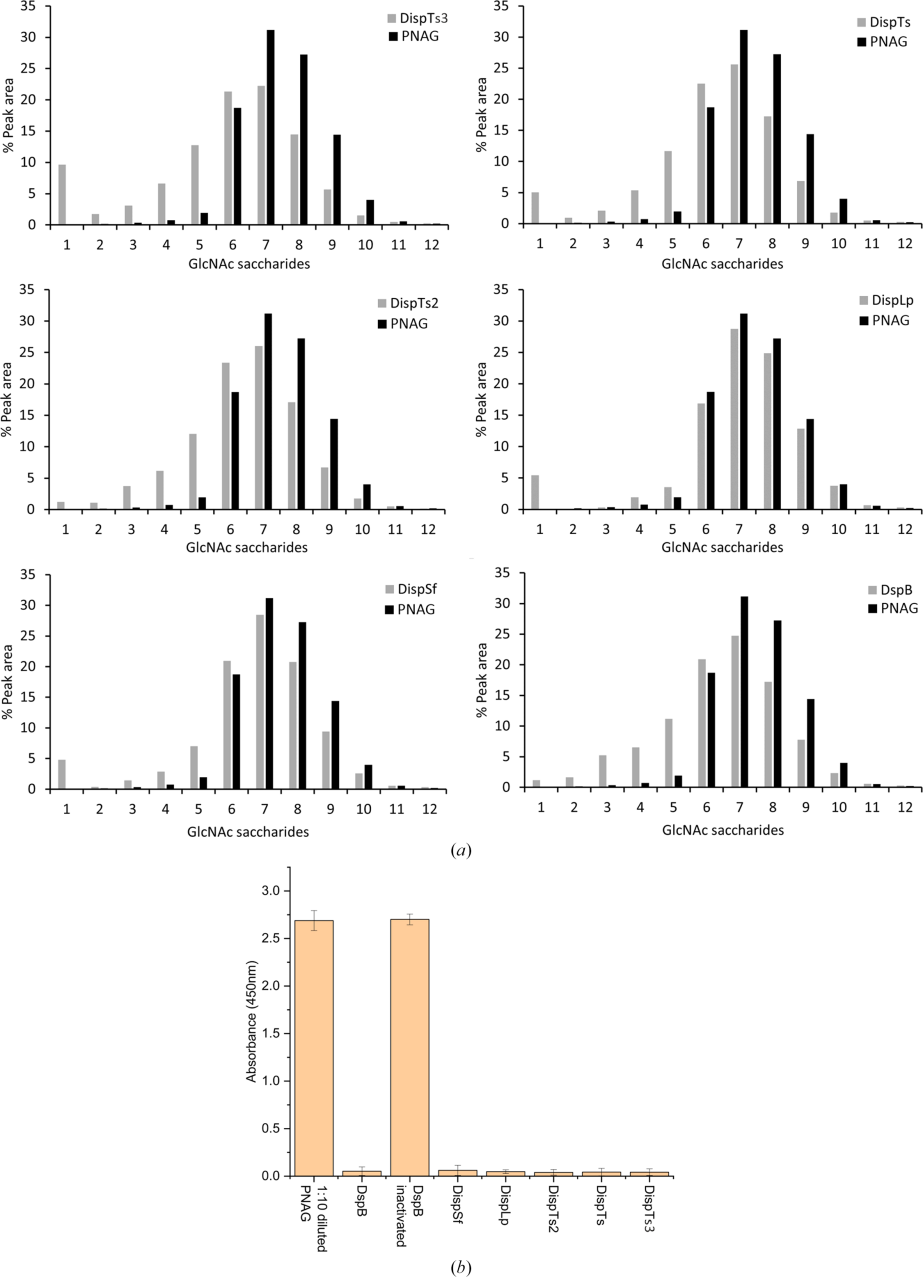
Activity of the putative dispersin enzymes on PNAG. (*a*) The enzymes were exposed to synthesized PNAG. After MALDI-TOF-MS data collection, the peak areas for each GlcNAc saccharide (including different adducts) were calculated. They were then totalled and converted to a percentage. (*b*) Indirect ELISA assay using crude PNAG from *P. fluorescens*.

Firstly, fully acetylated PNAG of varying lengths, mainly 6–10 GlcNAc units, was chemically synthesized using an acid-reversion reaction as previously reported and the high-molecular-weight fraction was isolated from size-exclusion chromatography (Leung *et al.*, 2009 ▸). The enzymes were incubated with the oligomer mixture overnight, the products were observed by MALDI-TOF-MS and the peak areas for each oligomer were compared (Fig. 3 ▸*a*). A clear trend towards a larger percentage of shorter PNAG saccharides (1–5 GlcNAc units) was seen upon incubation of the enzymes with PNAG. DispLp showed the lowest activity compared with the other dispersin enzymes, consistent with the activity data on *p*NP-GlcNAc/4MU-GlcNAc. This could be due to the lack of deacetylation that could be required for optimized binding of the substrate in preferred subsites of the active site. An intense monosaccharide peak (≥5%), perhaps inferring a preferentially exo-acting enzyme, is seen for DispTs3, DispTs, DispLp and DispSf. In contrast, less than 1.3% of the sugars hydrolysed by DispTs2 and DspB were monosaccharides, indicating a preference for acting in an endo manner.

Secondly, to verify that the enzymes showed activity on a natural, partially deacetylated, substrate, microbially derived PNAG was purified from *P. fluorescens* and exposed to enzymatic digestion. Activity was measured by indirect ELISA using an anti-PNAG primary antibody. As seen in Fig. 3 ▸(*b*), the novel dispersin enzymes and DspB showed activity on the natural substrate. In comparison to the synthetic PNAG substrate, DispLp showed increased activity on the partially deacetylated natural substrate, suggesting a greater preference for partially deacetylated substrates.

### 3.4. Crystal structures of the new dispersins

To understand the sequence conservation amongst the dispersins, and to better understand the key −1 subsite of these enzymes, which has so far evaded structural dissection, crystal structures of DispTs3, DispTs2 and DispLp were obtained (using seeding methods as discussed in Section 3) at resolutions from 2.0 to 1.05 Å (Supplementary Fig. S1 and Table 2 ▸). DispTs3, DispTs2 and DispLp all consist of a single domain with the expected (β/α)_8_ (TIM)-barrel fold for GH20 catalytic domains (Banner *et al.*, 1975 ▸; Tews *et al.*, 1996 ▸). The β-strands in the centre form a tunnel atop of which the active site is located in a groove, presumably to allow long chains of PNAG to bind. Superposition of the three dispersin structures and DspB revealed five areas which differed in secondary structure. Several α-helices within the outer ring of DspB are present as unstructured loops in the new dispersins; in contrast, loops within DspB have secondary structure in the dispersin variants (Supplementary Fig. S1 and Table S1). The β-strands β3 (Val75–Gly77), β4 (Gly80–Asn84) and β5 (Gly88–Pro90) are absent in DispTs3, DispTs2 and DispLp. On the opposite side of the protein to β3, β4 and β5 is an α-helix, α7 (Lys246–Met255), in DspB and DispLp; however, this region is present as two short β-strands in DispTs3 and DispTs2. The extra helices and loops present in DspB, and not in the new dispersins, reduce the length of the active-site groove, suggesting that it could be active on shorter PNAG substrates while the other dispersins may be active on longer substrates.

Since DispTs3 and DispTs2 have the highest sequence identity, as expected they have a small r.m.s.d. of 0.63 Å. In comparison, the low sequence identity between DispLp and both DispTs3 and DispTs2 resulted in larger differences; DispTs2 and DispLp have an r.m.s.d. of 1.67 Å and DispTs3 and DispLp have an r.m.s.d. of 2.30 Å.

No X-ray structures were obtained for DispTs, DispSf and DispCo, but *AlphaFold*2 predictions (Jumper *et al.*, 2021 ▸) resulted in structures similar to the dispersins discussed above, with the most significant differences for DispCo, which has an additional N-terminal domain, with the closest structures being fibronectin III type (FN3) domains, as identified by *GESAMT* (Krissinel, 2012 ▸; Supplementary Fig. S2). The relative orientation of the domains is likely to be correct based on the predicted aligned error (PAE) plot, where the blue colour of the regions corresponding to connection between residues from the catalytic and N-terminal domains (adjacent to the upper right and lower left corners of the plot) indicates high confidence of the relative positions of the domains (Supplementary Fig. S2*b*; Varadi *et al.*, 2024 ▸).

### 3.5. Complexes with 6-Ac-Cas provide insight into the active centre of dispersins

**Figure 4:**
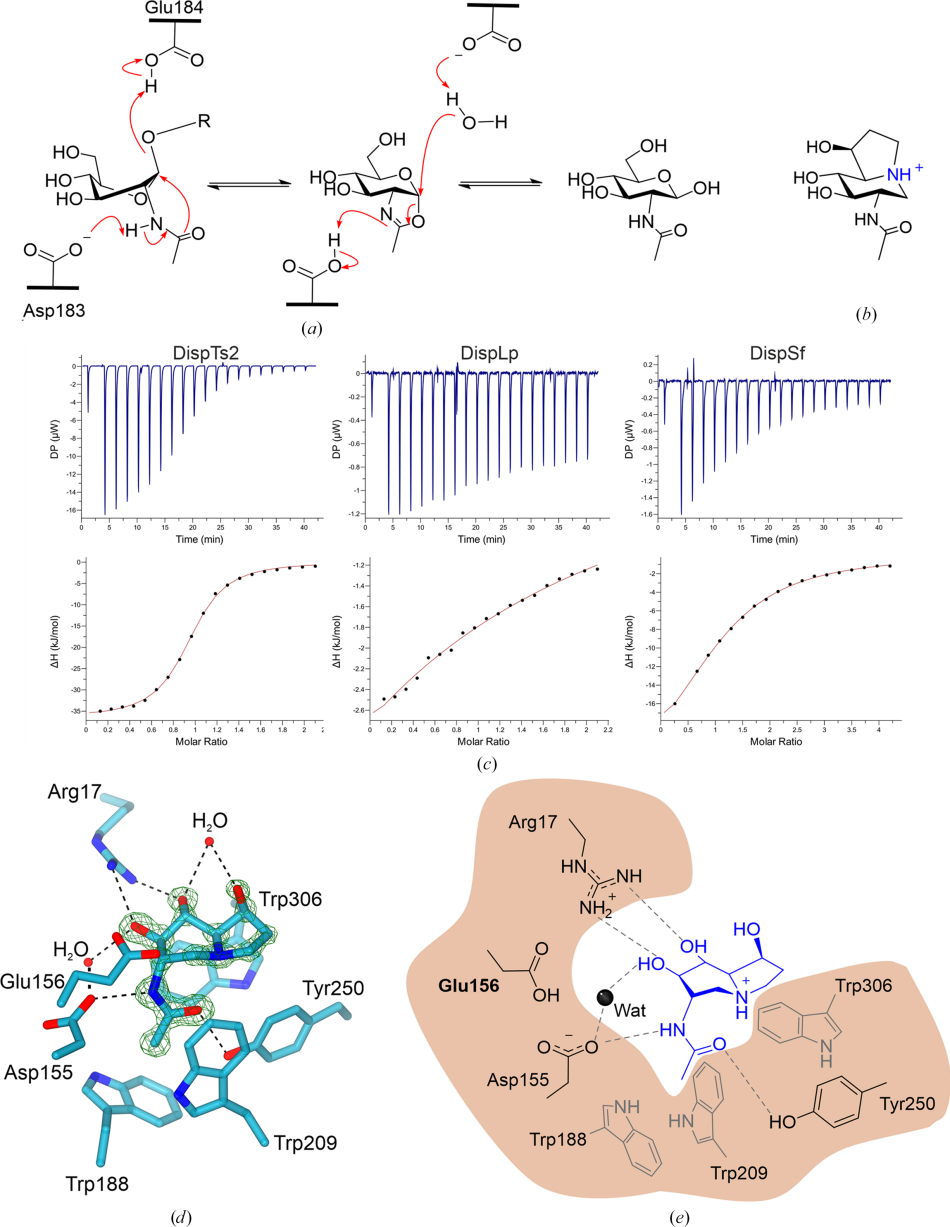
Catalytic mechanism and structure of 6-acetamido-6-deoxy-castanospermine. (*a*) Neighbouring-group participation catalytic mechanism of DspB. Here, we show the intermediate as a charged oxazolinium ion as predicted from calculations on related systems (Calvelo *et al.*, 2023 ▸). (*b*) Structure of 6-Ac-Cas. (*c*) Dispersin inhibition by 6-Ac-Cas. Thermodynamics of binding: the raw data are shown in the baseline-adjusted injection profile (top) and the titration curve with one-site fitting in red (bottom). Left: DispTs2, 0.93 ± 0.004 sites, −36.7 ± 0.2 kJ mol^−1^. Middle: DispLp, 0.90 ± 1.36 sites, −19.1 ± 0.35 kJ mol^−1^. Right: DispSf, 1.1 ± 0.02 sites, 26.4 ± 1.11 kJ mol^−1^. (*d*) Active-site residues of DispLp and water molecules with 6-Ac-Cas in complex. Hydrogen bonds are represented by dashed black lines and the maximum-likelihood/σ_A_-weighted 2*F*_obs_ − *F*_calc_ map is shown in green contoured at 0.90 e Å^−3^. (*e*) Scheme of DispLp active-site residue interactions with 6-Ac-Cas.

GH20 enzymes use a substrate-assisted catalytic mechanism, also referred to as neighbouring-group participation (NGP), in which the reaction proceeds via the formation and subsequent breakdown of a neutral oxazoline intermediate (Tews *et al.*, 1996 ▸; Drouillard *et al.*, 1997 ▸; Mark *et al.*, 2001 ▸). The acetamido group of the substrate acts as the nucleophile and a glutamate residue acts as the general acid/base (in this example Glu184 in DspB; Fig. 4 ▸*a*). The first insights into substrate distortion and catalysis were provided by studies of the *S. marcescens* chitobiase in complex with chitobiose (a disaccharide of β-1,4-linked GlcNAc; Tews *et al.*, 1996 ▸; Drouillard *et al.*, 1997 ▸). Whilst the catalytic mechanism is conserved for GH20 dispersins, there has been no information on the mode of ligand binding, with only a glycerol present in the published 3D structure. In order to gain insight into the dispersin active site, we first sought an inhibitor that would be amenable to structural analysis.

The use of iminosugars, which contain a substituted nitrogen in place of the ring oxygen, has provided important mechanistic insights into glycoside hydrolases. 6-Acetamido-6-deoxy-castanospermine (6-Ac-Cas), a derivative of castano­spermine which has a fused 5,6-indolizine ring system, is specific towards enzymes that use neighbouring-group participation and features an acetamido group introduced at the C2 position of the glucopyranose ring (Fig. 4 ▸*b*; Liu *et al.*, 1991 ▸). Three members of the GH20 family, the β-*N*-acetylhexosaminidase HexA from *Streptomyces coelicolor* A3(2) (*Sc*HexA; PDB entry 4c7f; Thi *et al.*, 2014 ▸), the β-hexosaminidase Hex1T from *Paenibacillus* sp. TS12 (PDB entry 3suw; Sumida *et al.*, 2012 ▸) and a lacto-*N*-biosidase from *Bifidobacterium bifidum*(PDB entry 5bxs; Hattie *et al.*, 2015 ▸), as well as a similar neighbouring-group participating family GH84 enzyme from *Bacteriodes thetaiotaomicron* (PDB entry 2xj7; Macauley *et al.*, 2010 ▸), have previously been crystallized in complex with 6-Ac-Cas in the −1 subsite. Therefore, we sought to determine whether 6-Ac-Cas was a suitable inhibitor for investigating the mechanism of the dispersin subfamily.

Binding constants for 6-Ac-Cas against a selection of dispersin enzymes were determined by isothermal titration calorimetry (ITC; Fig. 4 ▸*c*). 6-Ac-Cas has micromolar affinity towards DispTs2 and DispSf, with a *K*_d_ of 6 and 15 µ*M*, respectively. This is similar to literature values for other GH20 enzymes; 6-Ac-Cas with a GH20 exo-β-*N*-acetylhexosamini­dase from *Vibrio harveyi* had a *K*_d_ of 12.9 µ*M* (Meekrathok *et al.*, 2018 ▸). In marked contrast, the *K*_d_ of DispLp for 6-Ac-Cas was 1.12 m*M*.

Enzyme–inhibitor complexes were obtained with DispTs2 at a resolution of 1.51 Å and DispLp at a resolution of 1.05 Å after soaking the crystals in a solution containing 6-Ac-Cas, which we showed to be a potent inhibitor (Table 2 ▸). In the active site of both enzymes 6-Ac-Cas was distorted into a ^1^*S*_3_ conformation, which is consistent with the proposed catalytic pathway of GH20 enzymes based upon the ^1^*S*_3_/^4^*E* (Michaelis complex/product) conformation for GH20 enzymes that was first observed for the *S. marcescens* chitobiase (Tews *et al.*, 1996 ▸).

6-Ac-Cas was bound into the highly negatively charged −1 subsite notably via aspartate, glutamate and tyrosine residues (Figs. 4 ▸*d* and 4 ▸*e* and Supplementary Fig. S3*c*). The *N*-acetyl group is positioned in a hydrophobic pocket within the β-barrel. The acetamido carbonyl oxygen of 6-Ac-Cas is within hydrogen-bonding distance of the amine moiety of the indolizine ring at 2.56 and 2.61 Å in the active sites of DispTs2 and DispLp, respectively (Figs. 4 ▸*d* and 4 ▸*e*). Two key residues are involved in the NGP mechanism: a glutamate residue acts as the general acid/base (Glu184 in DspB, for example) and an aspartate residue deprotonates the *N*-acetamido group (Asp183 in DspB) (Fig. 4 ▸*a*). The catalytic glutamate residues, Glu161 in DispTs2 and Glu156 in DispLp, are 3.4 and 3.6 Å away from the anomeric carbon of 6-Ac-Cas, respectively, consistent with closer positioning to the glycosidic oxygen during catalysis. The position is stabilized by an interaction with His93 (DispTs2) and His94 (DispLp). A water molecule is poised for attack of the anomeric carbon at hydrogen-bonding distance to Glu156 (DispLp; Supplementary Fig. S4). Consistent with the key role of this glutamate, the E184Q variant of DspB lost its functionality (Manuel *et al.*, 2007 ▸). The catalytic aspartate, Asp160 in DispTs2 and Asp155 in DispLp, interacts with the NH group of the *N*-acetamido moiety, as required for the mechanism (Figs. 4 ▸*d* and 4 ▸*e*). A second water molecule in the active site is coordinated to the catalytic aspartate and O3 of 6-Ac-Cas. Mutation of the aspartate to an alanine in a GH20 β-hexosaminidase from *Streptomyces plicatus* resulted in the observation of the 2-acetamido group in two conformations, with only one of these being viable for catalysis (Williams *et al.*, 2002 ▸) and a 13 333-fold reduction in the catalytic efficiency of *p*NP-GlcNAc hydrolysis (Manuel *et al.*, 2007 ▸). Glu161 and Asp160 of DispTs2 are ∼5.2 Å apart, confirming that Glu161 is the general ‘glycosidic’ acid/base in catalysis.

A further three residues form hydrogen bonds to the ligand 6-Ac-Cas to facilitate ligand conformational changes, specifically to stabilize the transition-state conformation (Figs. 4 ▸*d* and 4 ▸*e*). A tyrosine, Tyr247 (DispTs2) and Tyr250 (DispLp), hydrogen-bonds to the oxygen of the *N*-acetyl group. The *N*-acetyl carbonyl group acts as the nucleophile during catalysis and the aspartate and tyrosine residues assist in polarizing and orientating the group (Williams *et al.*, 2002 ▸). Interestingly, in the structure of unliganded DispTs3 an acetic acid solute molecule was present in a similar position to the *N*-acetyl group of the GlcNAc. The acetic acid also formed hydrogen bonds to Asp160 and Tyr247 with distances of 2.55 and 2.75 Å, respectively (Supplementary Fig. S3*d*). Arg13 of DispTs2 (and likewise Arg17 from DispLp) forms two hydrogen bonds to the C3 and C4 hydroxyls of 6-Ac-Cas with distances of approximately 2.8 Å. Previous analysis of the importance of the arginine (Arg27) from DspB in ligand stabilization was analysed by mutating the residue to either alanine or lysine, which reduced the catalytic efficiency of *p*NP-GlcNAc cleavage by 1714-fold and 2400-fold, respectively, compared with the WT DspB enzyme when analysed by absorbance at 405 nm (Manuel *et al.*, 2007 ▸). Glu300 of DispTs2 forms two hydrogen bonds to the C4 hydroxyl, at a distance of 2.7 Å, and to the C6 hydroxyl on the pyrrole ring, at a distance of 2.75 Å. Mutation of the equivalent Glu332 of DspB to glutamine reduced the catalytic efficiency of *p*NP-GlcNAc hydrolysis by 2000-fold compared with the WT (Manuel *et al.*, 2007 ▸). Unusually, DispLp has an alanine instead of a glutamate at this position. This substitution could explain the 184-fold reduction in the dissociation constant of DispLp for 6-Ac-Cas compared with DispTs2 and 6-Ac-Cas. Therefore, distortion of 6-Ac-Cas in the active site of DispLp must rely on the interactions with Arg17 and Trp306. Interestingly, there are two waters in the DispLp structure that superpose well with the OE1 and OE2 of glutamate (Glu332 in DspB and Glu300 in DispTs2), one of which is coordinated by Gln252 (corresponding to Leu or Val in the other two dispersins); these waters might compensate for the Glu/Ala substitution (Supplementary Fig. S4).

Aromatic residues in the active site are involved in positioning the ligand correctly in the active site. A tryptophan residue, Trp298 (DispTs2) or Trp306 (DispLp), at the base of the active site provides important π–π stacking interactions through alignment of the indolizine rings of the tryptophan and the ligand. A second tryptophan, Trp193 in DispTs3 (Trp193 in DispTs2 and Trp188 in DispLp) is present at the base of the *N*-acetyl group. A third tryptophan, Trp214 in DispTs3 (Trp214 in DispTs2 and Trp209 in DispLp) forms the side hydrophobic pocket in which the *N*-acetyl group is situated; mutation of the corresponding DspB residue, W237A, completely abolished all detectable activity on *p*NP-GlcNAc, suggesting that the hydrophobic pocket is essential to capture the substrate (Manuel *et al.*, 2007 ▸).

The active-site pocket of all three dispersin enzymes and DspB is not as deep or enclosed as that of exo-acting GH20 enzymes. For example, the hexosaminidase from *S. plicatus* (*Sp*Hex), which has only exoglycosidase activity, has two unstructured loops, Thr272–Phe278 and Asp401–Tyr411, that lie on opposite sides of the active site, and which confine the top of the active-site pocket, restricting the enzyme to exo activity only (Mark *et al.*, 2001 ▸; Little *et al.*, 2012 ▸). In comparison, the cleft in which PNAG would bind to the three dispersins is shallow and could allow both endo and exo activity.

### 3.6. Complex of DispTs2 with GlcNAc-β(1,6)-GlcNAc-thiazoline

In order to trap a longer oligosaccharide complex, and building on the known neighbouring-group reaction mechanism, a novel disaccharide was synthesized (initial attempts with GlcNAc-thiazoline alone had not yielded high diffraction-quality crystals). This compound includes an additional β-1,6-linked GlcNAc to the well known GlcNAc-thiazoline, a potent transition-state/intermediate mimic and inhibitor of GH20 and related enzymes (Mark *et al.*, 2001 ▸; Macauley *et al.*, 2005 ▸; Knapp *et al.*, 1996 ▸). However, the design and synthesis, detailed in the supporting information, was performed before we had in-depth knowledge of the −2 subsite requirements of these enzymes.

**Figure 5:**
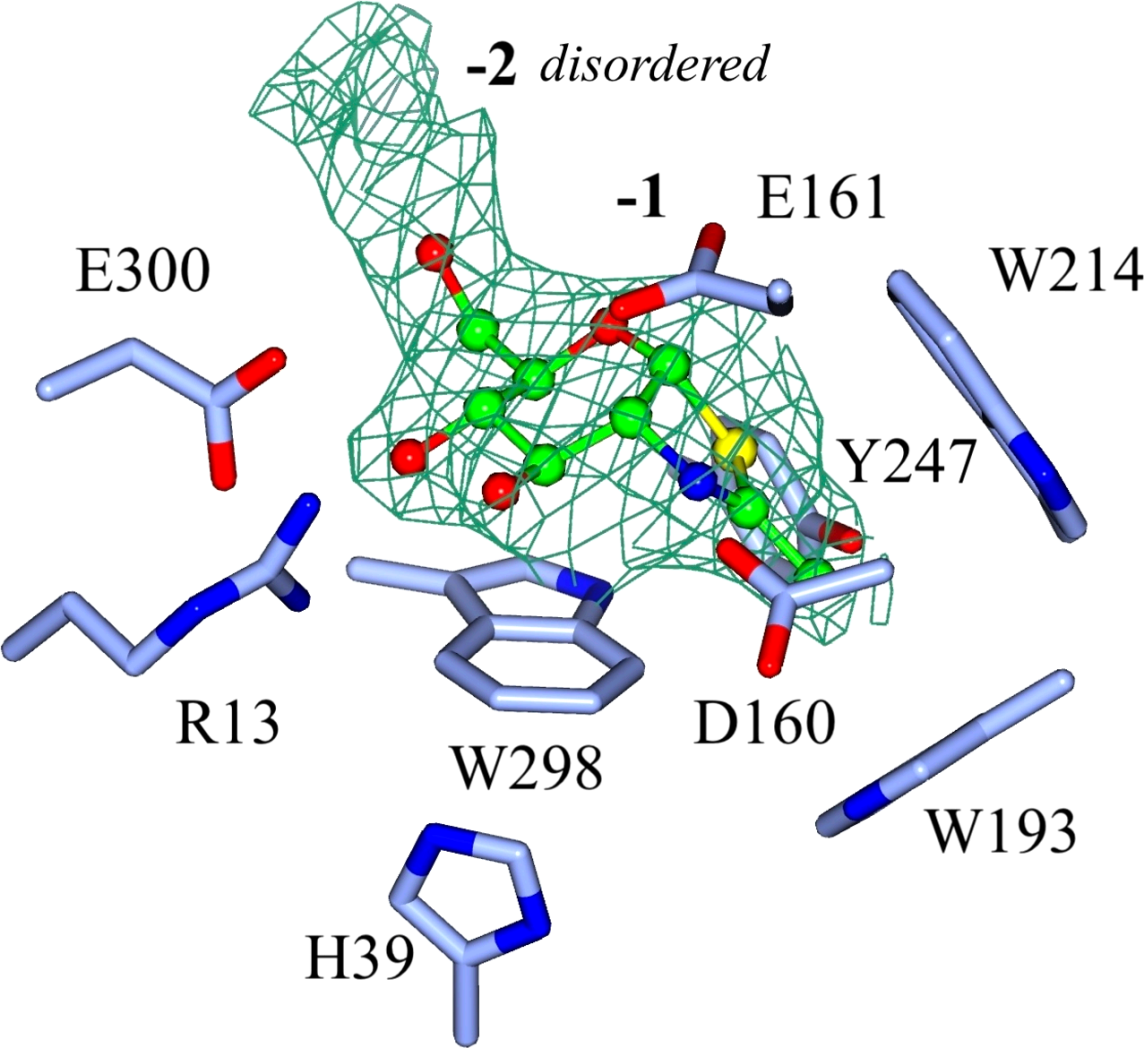
Structure of the active site of the complex of DispTs2 with di-NAG-thiazoline. The second GlcNAc (not shown) is not well defined, most probably because the −2 subsite preferentially accommodates GlcN rather than GlcNAc. The −1 and −2 subsites are shown in bold. The maximum-likelihood/σ_A_-weighted 2*F*_obs_ − *F*_calc_ map is shown in green contoured at 0.16 e Å^−3^. We did not create a new ligand library for this case because of poor density fit of the second unit; NAG-thiazoline only (NGT in the PDB ligand library) was modelled into the structure, shown in green.

We therefore conducted soaking experiments with the bespoke β−GlcNAc-β(1,6)-GlcNAc-thiazoline (di-NAG-thiazoline; see the supporting information for synthesis details). While these soaks produced crystals with poorer diffraction compared with 6-Ac-Cas, this compound held particular interest due to the additional sugar unit. The electron density for the first unit of di-NAG-thiazoline (corresponding to GlcNAc-thiazoline, NGT in the PDB dictionary) was well defined, reflecting its mimicry of the reaction intermediate, but only disordered density was observed for the −2 subsite. As subsequently discovered, the −2 subsite preferentially accommodates GlcN rather than GlcNAc, which likely contributed to the observed disorder of the GlcNAc moiety in this complex (Fig. 5 ▸).

### 3.7. The dispersins have signature conserved regions despite low sequence identities

**Figure 6:**
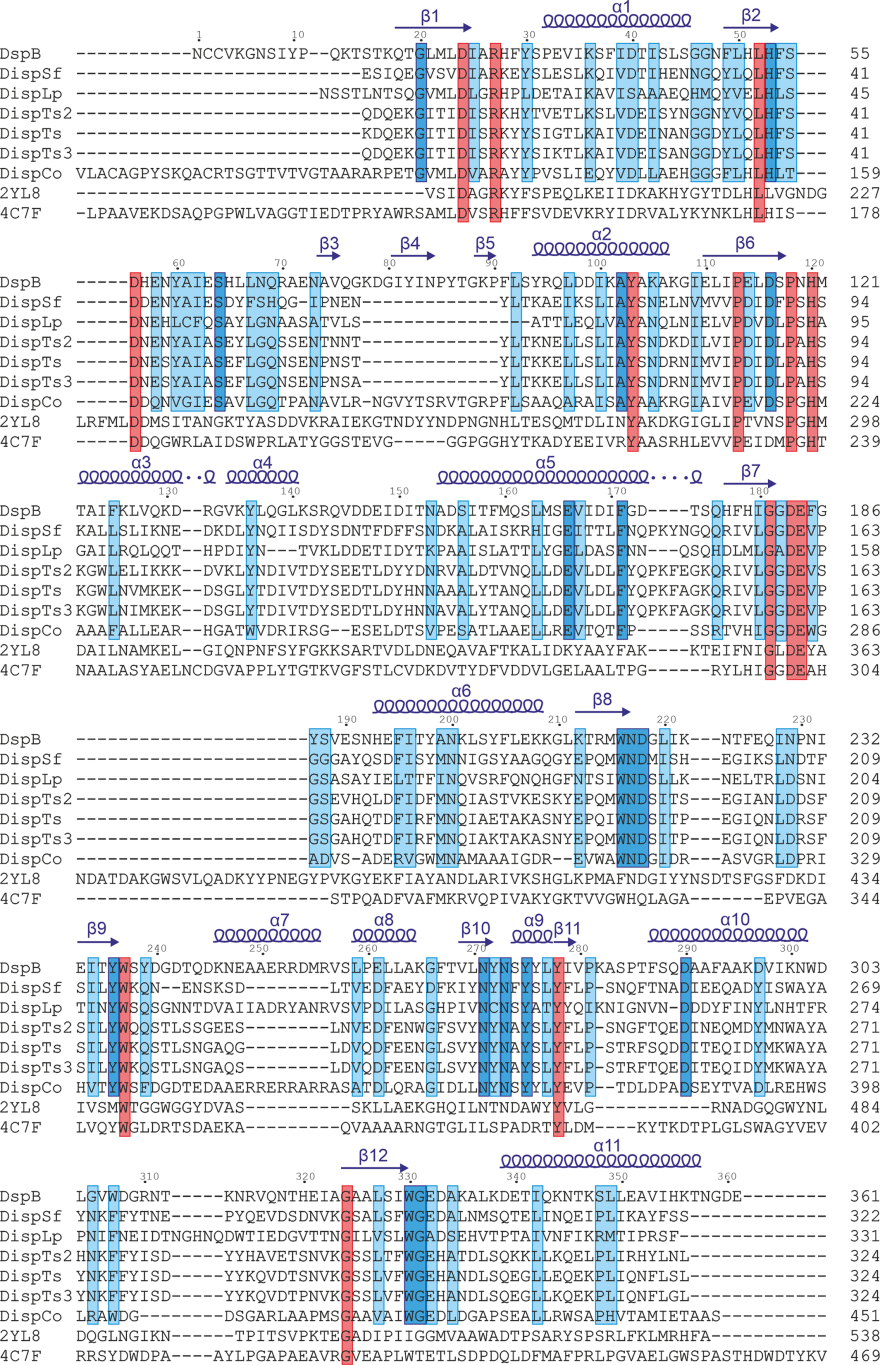
Sequence alignment of the PNAG catalytic domain of GH20 family members: DspB, the newly characterized dispersins, StrH (PDB entry 2yl8) and *Sc*HexA (PDB entry 4c7f). Residues with a red background are conserved across all GH20 proteins. Residues with a dark blue background are conserved across all dispersins. Residues with 70% conservation across dispersins are highlighted in a light blue box. The numbering and the secondary-structure elements across the top of the alignment correspond to the sequence and fold of DspB: α, α-helix; β, β-strand. For DispCo, the N-terminal domain was omitted; the first residue included was Val102. In PDB entry 2yl8, the catalytic residue Glu361 of StrH is mutated to a glutamine; only the GH20 domain from residues 190 to 538 was used in the alignment. For *Sc*HexA (PDB entry 4c7f), the GH20 domain between residues 153 and 535 (the C-terminus) was used in the alignment. Domain boundaries were predetermined (Val-Cid *et al.*, 2015 ▸).

Having demonstrated that these enzymes were all hexosaminidases active on PNAG and obtained the crystal structures, we next sought to analyse any sequence features that were conserved amongst the dispersins and to map them onto the 3D structure to aid future dispersin categorization. All of the putative dispersins were not previously members of CAZy family GH20 (Lombard *et al.*, 2014 ▸); therefore, a sequence alignment with DispB, a single-domain β-1,4 *N*-acetyl­glucosaminidase (StrH) from *S. pneumoniae* TIGR4 and the representative multi-domain *Sc*HexA was performed (Fig. 6 ▸).

**Figure 7:**
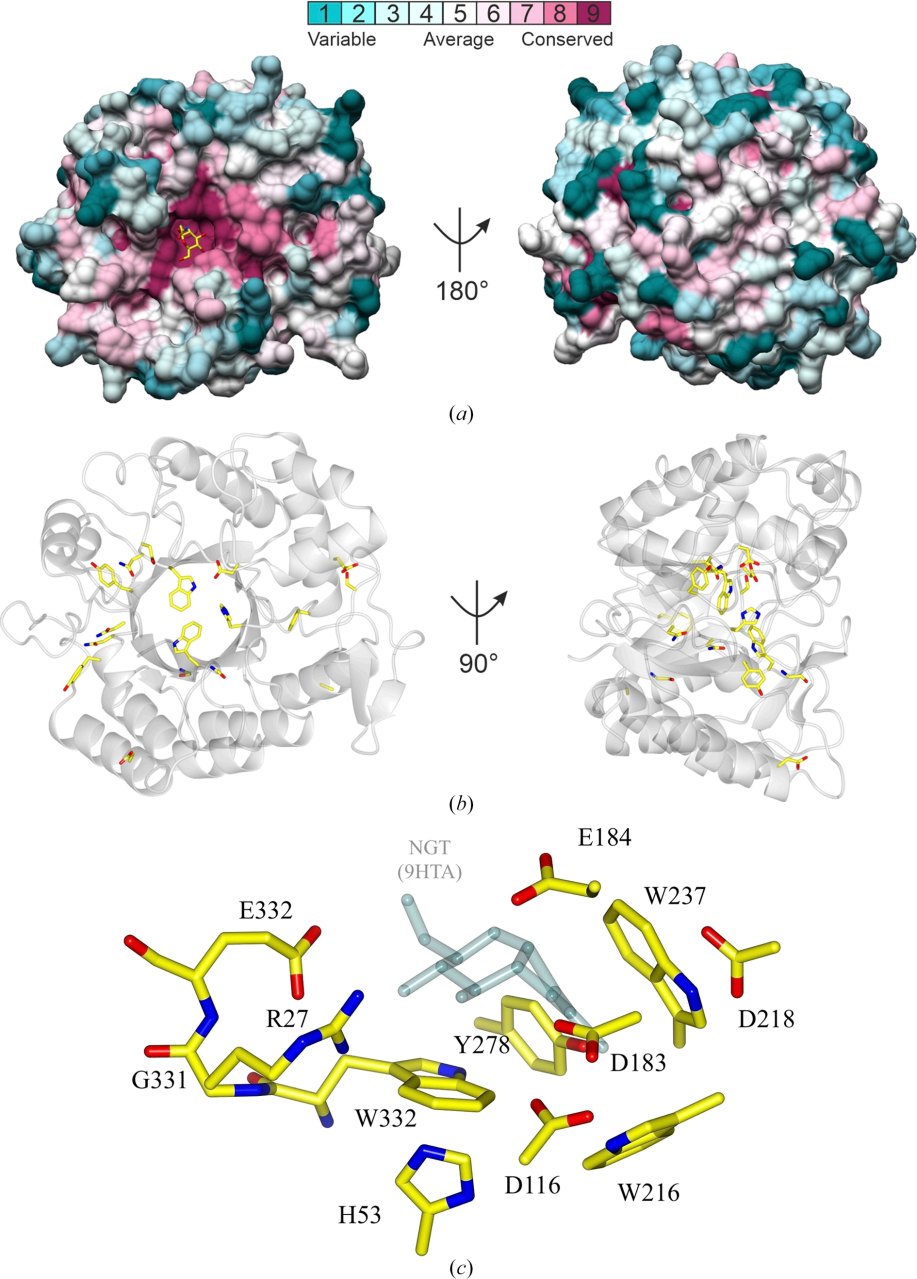
Conserved residues in GH20 enzymes are clustered in the active site and on the top face. (*a*) Surface representation of DspB (PDB entry 1yht) in white with residues coloured according to the degree of sequence conservation using the aligned proteins from Fig. 1 ▸. GlcNAc, in yellow, was modelled into the active site by superimposing DspB (PDB entry 1yht) with a β-*N*-acetylhexosaminidase from *Akkermansia muciniphila* (PBD entry 7cbo; Xu *et al.*, 2020 ▸). This figure was produced using the *ConSurf* server (Landau *et al.*, 2005 ▸; Ashkenazy *et al.*, 2016 ▸). (*b*) Ribbon representation of DspB (PDB entry 1yht) with conserved residues specific to the dispersin subfamily members (not conserved in other GH20 enzymes) highlighted in yellow. (*c*) A close-up view of the dispersin active site in the example of DispB (PDB entry 1yht), with key residues numbered for DspB. NAG-thiazoline (NGT) from the DispTs2–di-NAG-thiazoline complex structure (PDB entry 9hta), in semi-transparent grey, is shown to indicate the ligand-binding site.

The regions of high sequence conservation are situated within the active site and on the top face of the enzyme, and 14 residues (Fig. 7 ▸*a*) that are conserved across both GH20 single-domain and multi-domain enzymes are located facing inwards towards the centre of the barrel (Fig. 7 ▸*b*). Of these 14, eight residues are conserved across all GH20 enzymes analysed and six residues are only conserved across the dispersin subfamily (His53, Asp116, Trp216, Asp218, Trp330 and Gly331 of DspB; Fig. 7 ▸*c*). The *N*-acetyl group of GlcNAc in the −1 subsite is surrounded by three tryptophan residues that form a compact hydrophobic pocket. Tyr237, which is located at the side of the active-site pocket against the *N*-acetyl group, is conserved across all GH20 enzymes; however, Trp330 and Trp216 are specifically conserved in all dispersin enzymes. His53 is located at the base of the active site perpendicular to Trp330. Asp116 is at hydrogen-bonding distance from the catalytic residue Asp184, Asp218 is at hydrogen-bonding distance from Tyr237, and Gly331 is found between Trp330 and Glu332, which make important ligand interactions. These conserved residues in the dispersin subfamily are important for positioning and stabilizing key catalytic residues.

In the structure of DispTs2, Glu300 (Glu332 in DspB) forms important ligand interactions with the C4 and C6 hydroxyls of the pyrrole ring to stabilize the ligand conformational changes during catalysis. This residue is conserved in all dispersins apart from DispLp (Ala308) and the equivalent residue is a glycine in StrH. Therefore, as well as implications for its catalytic efficiency attributed to a loss in hydrogen-bonding capacity, DispLp might be able to accept a β-1,4-linked substrate since there would not be any steric clashes from the 4-position, with the groove now 3 Å larger.

A further 11 residues are conserved throughout the dispersin subfamily. Gly20 is located in the central β-barrel (β1); Ser64 (loop between β2 and β3) and Glu166 (α5) form a hydrogen bond; Ala102 (α2) is a surface residue; Phe171 (α5) forms stacking interactions against Pro113 (β6), which is conserved across the GH20 family; Asn217 (the loop after β8) is located between Tyr216 and Asp218 that play important roles in the active site and Tyr236 (β9) is located next to Asn217 in the structure; Asn271, Asn273 and Tyr275 (the loop between β10 and α9) stabilize the loop region between α10 and β12; and Asp290 (α10) is a surface residue.

The catalytic motif for GH20 enzymes, required for their NGP catalytic mechanism, consists of a catalytic aspartate and glutamate. In DspB, DispCo and *Sc*HexA, the DE motif is preceded by H*X***G**G(**DE**), whereas StrH contains the sequence NIGL**DE**. DispSf, DispTs2, DispTs and DispTs3 have the sequence VL**G**G**DE** and DispLp has the sequence ML**G**A**DE** (Fig. 6 ▸). Hence, there is no consistent sequence motif requirement for dispersin catalytic sites. The two main regions of sequence conservation between dispersins only are Trp216–Trp218 and Asn271–Tyr275, which are important in catalysis and loop stabilization, respectively.

## 4. Discussion

Under certain stresses and signals, microorganisms use different mechanisms to break the extracellular matrix for cell dispersion. Depending on the composition of the EPS, proteases, DNases and PNAGases are responsible for the release. Several new PNAG-cleaving dispersins have been identified and characterized in this study. The five novel dispersins examined were identified amongst taxonomically well separated bacterial genera, which could point to a common ancestral source. However, it cannot be excluded that these enzymes evolved independently on multiple occasions. Interestingly, there was no clear link to a specific ecological niche as the bacterial hosts originated from diverse sources. This could suggest that the activity is linked to a more fundamental microbial characteristic, such as dispersal (Penesyan *et al.*, 2021 ▸).

Around 50 additional enzymes were identified through a *BLAST* search based on sequence similarity to DspB. Further analysis of these enzymes, using the assays described in this paper, could expand the dispersin subfamily. The location of the DNA sequences compared with the sequences involved in forming the PNAG biosynthetic machinery could be important for further verification. Genes within the *pga*/*ica* operons could be identified in *T. saccharophilus* (*pgaC* and *pgaD*), *M. fleurettii* (*icaB*, *icaD* and *pgaC*) and *L. paraplantarum* (*pgaC*); however, neither the *pga* nor *ica* gene operons could be identified in *C. oceanosedimentum*. It cannot be excluded that this species, and the other species with only select genes in the cluster assigned, carry unassigned operons related to PNAG production. These enzymes could also be targeting other types of extracellular polysaccharides produced by the host cells. Another intriguing explanation could be that these enzymes impose a competitive or cooperative advantage in a polymicrobial environment. Bacterial species are known to interact actively in sessile communities (Burmølle *et al.*, 2014 ▸), forming predatory or symbiotic relationships depending on the species composition. It could be speculated that these PNAG-degrading enzymes, found in microorganisms without any apparent genes coding for PNAG production, could in fact serve as dispersal agents in multispecies communities where other PNAG producers are present, enabling the dispersin-producing microorganisms to compete or corroborate with their neighbours. It would have been of interest to carry out further studies on DispCo with the aim of obtaining a sample including the N-terminal domain allowing crystallization and kinetic measurements, but this was beyond the scope of this project. *AlphaFold*2 modelling of DispCo suggested that its N-terminal domain was fibronectin-like.

Obtaining the structure of new dispersins in complex with a ligand confirmed the substrate-assisted catalysis mechanism and revealed important residues involved in catalysis through positioning the substrate for catalysis and stabilizing the conformational changes along the reaction coordinate of the enzyme. The inhibitor 6-Ac-Cas is supported by a hydrogen-bonding network between residues in the active site of DispTs2 and DispLp and the dispersin-specific tryptophan residues which form the base of the active site.

The GH20 family contains enzymes that cleave a variety of different substrates, although these enzymes have high sequence identity. A small number of enzymes are chitobiases, which cleave the β-1,4-GlcNAc linkage of chitin, and lacto-*N*-biosidases, which cleave the β-1,3-linkage between GlcNAc and galactose (Tews *et al.*, 1996 ▸). The predominant type of enzymes in this family are β-hexosaminidases, acting on both *N*-acetyl­glucosamine and *N*-acetylgalactosamine. Primarily, these enzymes are not known to cleave PNAG, while a broader range of these enzymes cleave *p*NP-GlcNAc. All five of the dispersins tested showed measurable activity on both small-molecule substrates, *p*NP-Glc-NAc and 4-MU-GlcNAc, with DispTs2 as the most active on both substrates and DispLp as the least active on both substrates. Most notably, DspB is the dispersin that shows the largest difference in specificity towards the two substrates. This may be attributed to the small differences between the two substrates, where *p*NP-Glc-NAc is the smaller substrate and is able to display a small partial charge from the resonance structure of the –NO_2_group. Conversely, 4-MU-GlcNAc is slightly larger and displays a larger fused aromatic structure which is more polarizable. Recently, a new fluorogenic substrate has been developed which includes a carbamate linker between the GlcNAc and the fluorophore, 7-amino-4-methylcoumarin, to increase the distance and allow efficient hydrolysis (Wang *et al.*, 2022 ▸).

In conclusion, the activity of DispTs2 is comparable to that of DspB. Supplementary evidence for the location of the various subsites, the preference of these subsites for GlcNAc or GlcN from the surrounding residues, for example Asp147, Asp245 and Glu248 in DspB, and the ability of the dispersins to be both exo and endo acting require a crystal structure with a complex of a dispersin and a polysaccharide ligand. Moreover, the differences in the secondary structure that we observed between the different dispersins might have an impact on their exo/endo-acting propensities, which could be an interesting topic for future studies. Further research is also needed into the organization of the PNAG biosynthetic machinery in the cell envelope, in reference to the PgaABCD and IcaABCD enzymes, and the association of PNAG with itself and other components on the cell surface. Recently, a general acid/base, GFP-tagged mutant of DspB was used as a probe to detect PNAG oligomers in high-density and isolated regions, PNAG islands, on the periphery of the cell during the early log phase and extending between bacteria as the point of contact (Eddenden *et al.*, 2020 ▸).

It is hoped that the enzymes discussed in this paper will act as an alternative for using DspB to elucidate these questions. Whether they have superior function under alternate conditions or alternate immunoreactivity will need to be established. This work paves the way for the unearthing of additional dispersin enzymes.

## 5. Related literature

The following references are cited in the supporting information for this article: Fulmer *et al.* (2010 ▸), Jiang *et al.* (2004 ▸), Maiti *et al.* (2007 ▸) and Reynolds & Evans (1942 ▸).

## Supplementary Material

PDB reference: DispTs3, 8qak

PDB reference: DispTs2, 8qb6

PDB reference: DispLp, 8qce

PDB reference: DispTs2, complex with di-NAG-thiazoline, 9hta

Supplementary Table end Figures, Supplementary Methods and NMR spectra. DOI: 10.1107/S205979832500110X/rr5249sup1.pdf

## Figures and Tables

**Table 1:** Enzyme designations, corresponding bacterial strain sources and DNA accession numbers

Enzyme	Donor organism	GenBank ID
DispTs	*Terribacillus saccharophilus*	OM214561
DispTs2	*Terribacillus saccharophilus*	OM214562
DispTs3	*Terribacillus saccharophilus*	OQ858607
DispLp	*Lactiplantibacillus paraplantarum*	60A0A6M5IIS2
DispSf	*Mammaliicoccus fleurettii*	94A0A3A0I2R9
DispCo	*Curtobacterium oceanosedimentum*	OM214560
DspB	*Aggregatibacter actinomycetemcomitans*	AAP31025.1
PgaB	*Bordetella bronchiseptica*	CAE32265.1

**Table 2:** Data-collection statistics and structure-solution and refinement statistics Values in parentheses are for the outer shell.

	DispTs3	DispTs2 + 6-Ac-Cas	DispTs2 + di-NAG-thiazoline	DispLp + 6-Ac-Cas
PDB code	8qak	8qb6	9hta	8qce
Beamline	I03, DLS	I04, DLS	I03, DLS	I03, DLS
Wavelength (Å)	0.976	0.9795	0.976	0.976
Temperature (K)	100	100	100	100
Space group	*P*2_1_2_1_2_1_	*P*3_2_21	*P*3_2_21	*P*12_1_1
*a*, *b*, *c* (Å)	50.9, 109.4, 131.1	89.7, 89.7, 97.7	90.1, 90.1, 98.1	46.9, 82.8, 80.6
α, β, γ (°)	90, 90, 90	90, 90, 120	90, 90, 120	90, 98.1, 90
Completeness (%)	100 (100)	100 (100)	93.1 (76.4)	90.5 (40.3)
Multiplicity	4.9 (4.8)	10 (10)	11.6 (10.3)	3.7 (2.2)
*R*_p.i.m._ †	8.2 (81.5)	1.0 (62.8)	4.0 (215.1)	5.3 (93.5)
〈*I*/σ(*I*)〉	7.3 (1.2)	17.7 (1.2)	13.3 (0.5)	6.6 (0.7)
Resolution range (Å)	83.97–1.95 (2.0–1.95)	30.03–1.51 (1.54–1.51)	40.93–2.17	38.06–1.05 (1.07–1.05)
CC_1/2_‡	0.99 (0.34)	0.99 (0.51)	0.99 (0.53)	0.99 (0.42)
Final *R*_cryst_/*R*_free_	0.19/0.23	0.15/0.18	0.21/0.25	0.14/0.17
No. of non-H atoms (chain *A*/*B*)				
Protein	2602/2610	2672	2631	2710/2856
Ligand	—	16	14	16/16
Water	333	193	43	772
Solute	8 (ACT)	16 (MPD)	—	7 (PEG), 12 (EDO)
R.m.s. deviations				
Bond lengths (Å)	0.012	0.010	0.0055	0.011
Angles (°)	1.91	1.59	1.4820	1.73
Average *B* factors (Å^2^)				
Protein	36/31	28.7	60.1	13/17
Ligand	N/A	33.1	60.7	14/16
Water	32	36.0	54.5	32
Ramachandran plot				
Most favoured (%)	97	97.5	96.0	98.7
Allowed (%)	2.8	2.5	4.0	1.3
Outliers (%)	0.02	0	0	0

† *R*_p.i.m._ = 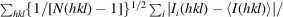

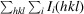
.

‡ CC_1/2_ is defined in Karplus & Diederichs (2012 ▸).
