# Supplementary material for: Expansion of the diversity of dispersin scaffolds
Source: Acta Crystallogr D Struct Biol. 2025 Feb 28;81(Pt 3):130–46. doi: 10.1107/S205979832500110X (PMC11883664; doi:10.1107/S205979832500110X)
Supplement: Supplementary file 1 [file d-81-00130-sup1.pdf]

STRUCTURAL BIOLOGY

Volume 81 (2025)

Supporting information for article:

Expansion of the diversity of dispersin scaffolds

Alexandra Males, Olga V. Moroz, Elena Blagova, Astrid Munch, Gustav H. Hansen, Annette H. Johansen, Lars H. Østergaard, Dorotea R. Segura, Alexander Eddenden, Anne V. Due, Martin Gudmand, Jesper Salomon, Sebastian R. Sørensen, João Paulo L. Franco Cairo, Mark Nitz, Roland A. Pache, Rebecca M. Vejborg, Sandeep Bhosale, David J. Vocadlo, Gideon J. Davies and Keith S. Wilson

Table S1 Differences in the secondary structure of the dispersins.

Discrepancy	DspB	DispTs3	DispTs2	DispLp
1	β3 V75-G77<br>β4 G77-N84<br>β5 G88-P90	UL N59-A63	UL N59-T63	UL S62-T64
2	UL between α4 and α5 R143-I149	β-strand T120-L121	β-strand T120-L121	β-strand K114-D116<br>β-strand T119-I120
3	UL K222-Q227	α-helix P199-N204	UL S199-N204	UL K194-R199
4	α7 K246-M255	β-strand T218-L219<br>β-strand G222-Q224	β-strand T218-L219<br>β-strand G222-E224	α-helix V218-N227
5	UL between α10 and β12 N301-G324	β-strand K274-F275<br>β-strand K283-Q284	β-strand K274-F275<br>β-strand H283-A284	β-strand I277-F278<br>β-strand E280-D282<br>β-strand G285-Q288<br>β-strand T291-I292

UL=Unstructured loop

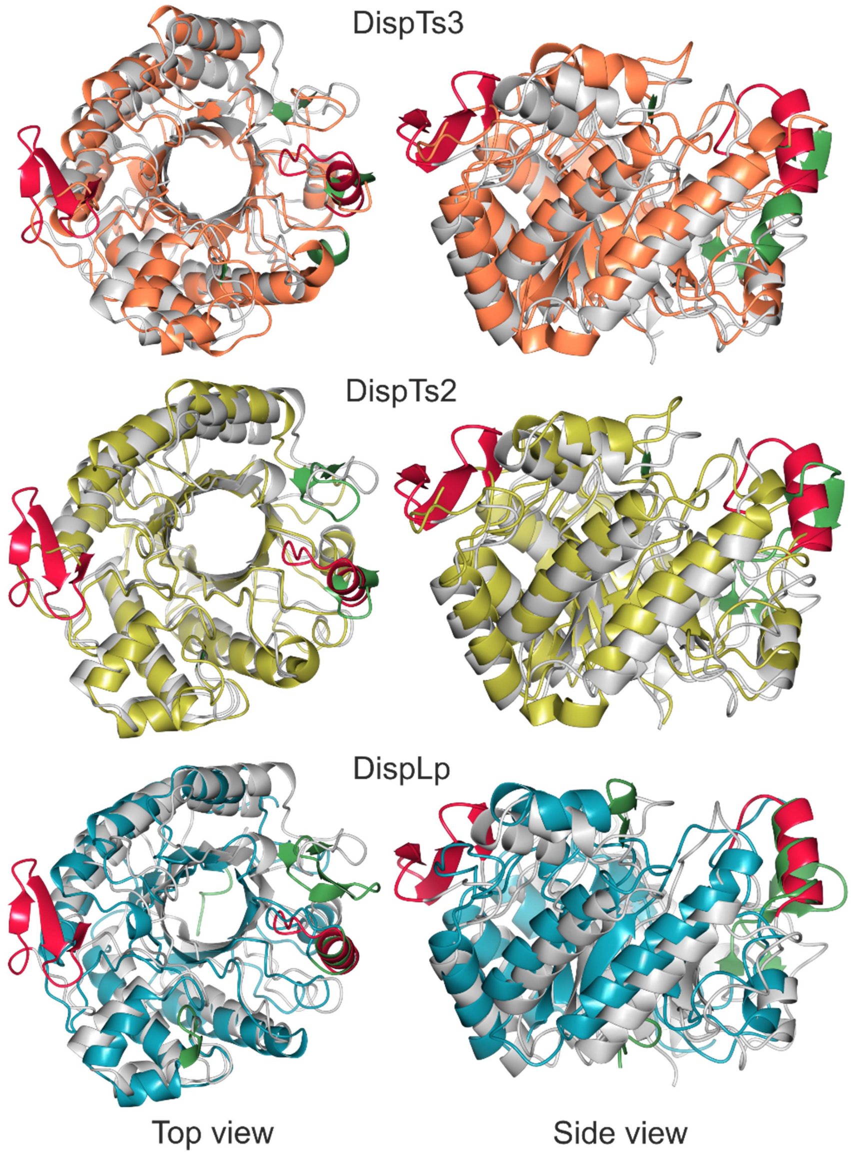

Figure S1 Comparison of secondary structure differences between DspB and DispTs3, DispTs2 and DispLp. Top view and side view of superpositions of the crystal structures of DspB (PDB ID:1YHT) shown in grey, DispTs3 shown in orange, DispTs2 shown in yellow and DispLp shown in blue. Secondary structure elements different between DspB and other dispersins are colour coded - for DispB these are shown in red, and for the other three dispersins they are in green.

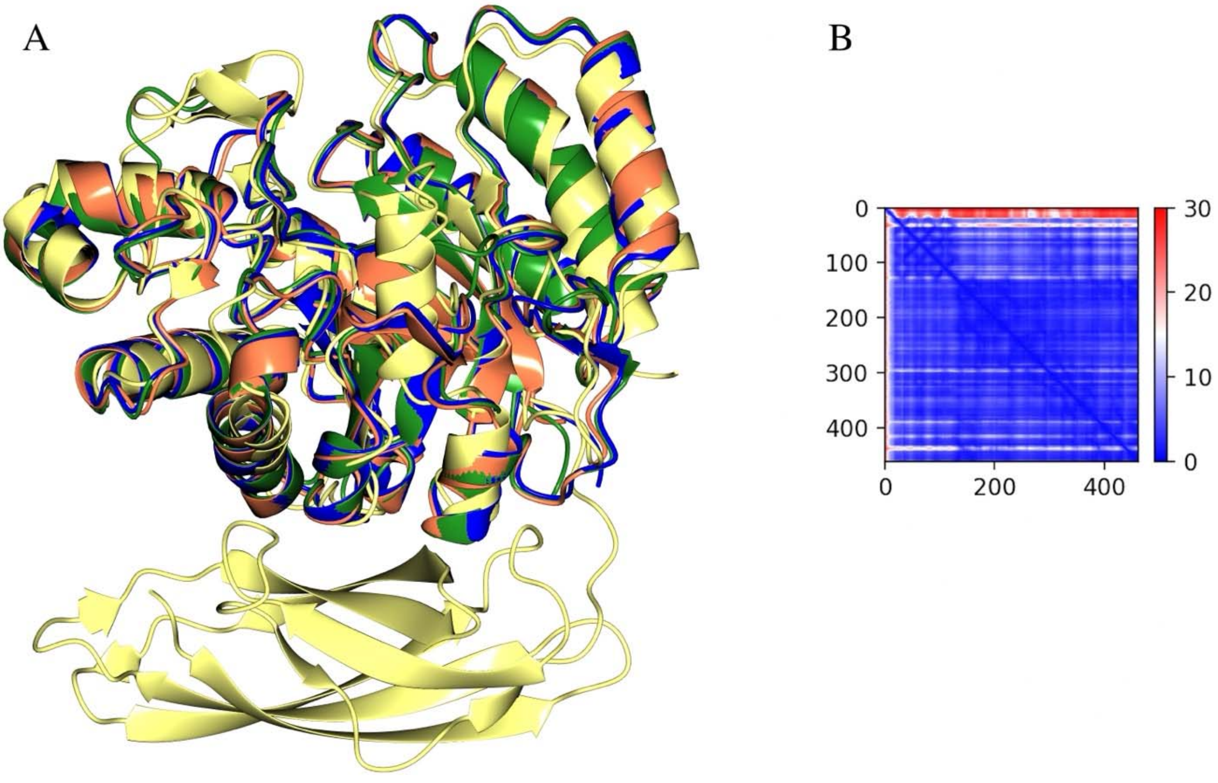

Figure S2 A) Structure superpositions of AF2 models for DispTs and DispSf, and DispCo on the X-ray structure of DispTs3. DispTs (shown in blue) is non-surprisingly the closest to Ts3 (rmsd 0.54Å, for 318 aligned residues), followed by DispSf (in green, 0.83Å/304 aligned residues). DispCo (in yellow, 1.42Å/286) has more differences in the catalytic domain, with two short beta strands in the region corresponding to 57-62 of DispTs3, and an extra alpha helix in the region corresponding to the loop 217-226. In addition, it has an N terminal domain with similarity to fibronectin type-III domains, identified by Gesamt (Krissinel, 2012). The DispCo structure shown starts at the beginning of the AF2 higher confidence region (pLDDT 70.0 onwards) , Arg42. B) PAE plot indicates high likelihood of relative positions of two domains of DispCo predicted by AF2 (Varadi *et al.*, 2023).

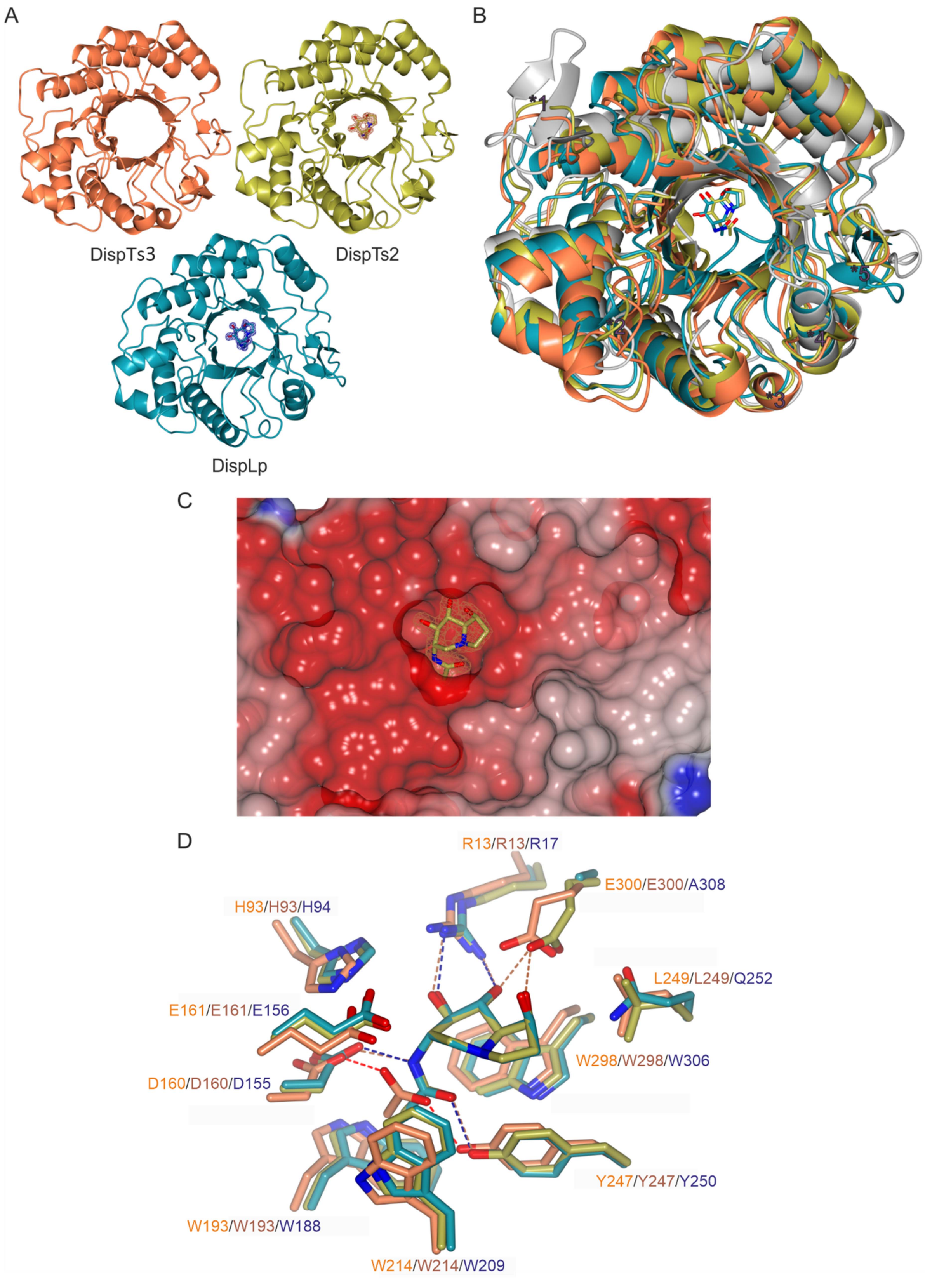

A
B
DispTs3
DispTs2
DispLp
C
D
R13/R13/R17
E300/E300/A308
H93/H93/H94
L249/L249/Q252
E161/E161/E156
W298/W298/W306
D160/D160/D155
Y247/Y247/Y250
W193/W193/W188
W214/W214/W209

Figure S3 The structures of DispTs3, DispTs2 and DispLp reveal a conserved active site primed for binding GlcNAc residues. The same colour theme is used throughout this figure; DispTs3 is shown in orange, DispTs2 in gold, DispLp in blue and DspB in grey. A) The overall $(\beta/\alpha)_8$ fold of the dispersin enzymes. In the active sites of DispTs2 and DispLp is 6-Ac-Cas with the maximum-likelihood/σ_A-weighted $2F_{obs} - F_{calc}$ map shown in gold or blue contoured at 0.64 e Å^{-3}. B) Superposition of the DispTs3, DispTs2, DispLp and DspB structures. C) Surface representation of the active site of DispTs2 with 6-Ac-Cas bound in a negatively charged pocket. D) Superposition of the residues within the active site of DispTs3, DispTs2, DispLp. Hydrogen bonds are indicated by coloured lines complementary to the colour of the residues. 6-Ac-Cas, shown in gold and blue, is bound in the active site of DispTs2 and DispLp, respectively. An acetate molecule, orange, is bound in the active site of DispTs3.

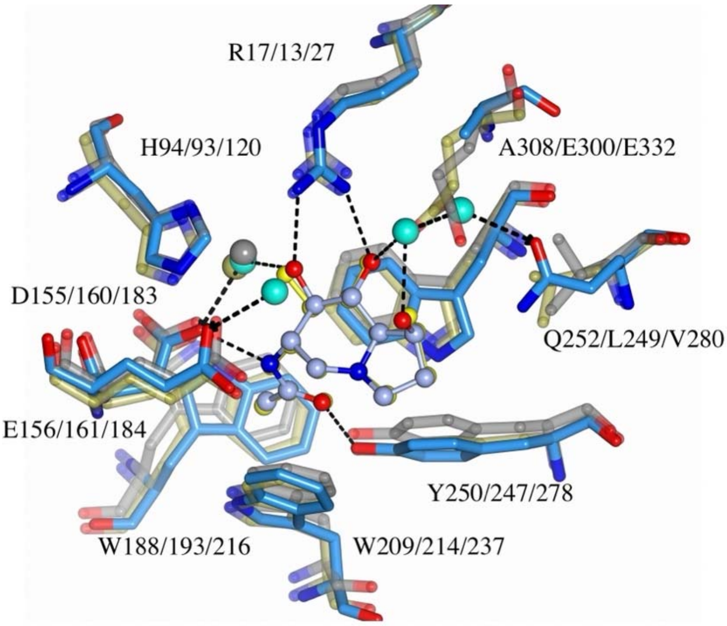

Figure S4 Stereo view of the superposition of DispLp, DispTs2 (both in complex with 6-Ac-Cas) and /DspB. DispLp is in blue, DispTs2 in gold and DspB in grey. Waters are cyan for DispLp, gold and grey for DispTs2 and DspB correspondingly. Two waters from DispLp superpose well with OE1 and OE2 of glutamates from two other dispersins, possibly to compensate for alanine instead of glutamate in DispLp, one of these waters is coordinated by Q252 (corresponding to Leu and Val in other two dispersins).

S1. Supplementary Methods

S1.1. Chemical synthesis and compound characterization

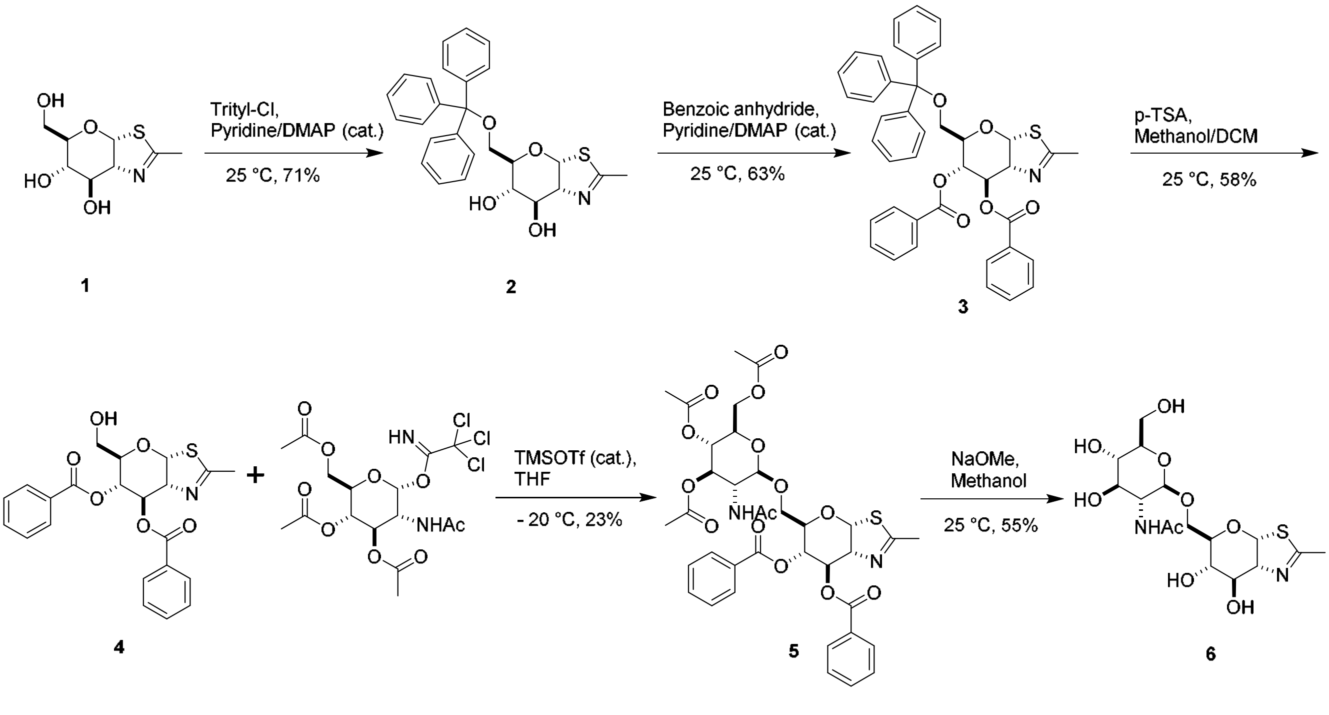

Scheme S1. Chemical synthesis.

Nuclear magnetic resonance (NMR) spectra were recorded on a Bruker Avance 600 equipped with either a QNP or a TCI cryoprobe (600 MHz), Bruker 500 (500 MHz), or Bruker 400 (400 MHz) at ambient temperature. NMR solvents d_6–acetone {$(CD_3)_2CO$} or Acetone-*d6*; *d*–chloroform ($CDCl_3$), d_4 –methanol (CD_3OD), deuterium oxide (D_2O), and anhydrous d_6-dimethylsulfoxide (DMSO-d_6) were purchased from commercial suppliers and used without further purification. Spectra were processed using the automatic phasing and Whittaker Smother baseline correction features of MestReNova software. Spectral data are reported as follows: chemical shift (multiplicity [singlet (s), broad singlet (bs), doublet (d), triplet (t), quartet (q), multiplet (m), doublet of doublets (dd), doublet of doublet of doublets (ddd), doublet of triplets (dt)], coupling constant, integration). Chemical shifts (δ) are listed in ppm downfield from TMS using the residual solvent peak as an internal reference, and coupling constants are reported in Hz. ^{1}H resonances are referenced to solvent residual peaks (Fulmer *et al.*, 2010). ^{1}H and ^{13}C NMR peak assignments are made based on ^{1}H-^{1}H COSY, ^{1}H-^{13}C HSQC (Heteronuclear Single Quantum Coherence), and HMBC (Heteronuclear Multiple Bond Correlation) experiments. Routine ^{13}C NMR spectra were recorded on a 500 or 600 MHz spectrometer with protons decoupled unless otherwise noted. ^{13}C resonances are reported in ppm relative to solvent

residual peaks (Fulmer *et al.*, 2010). High-resolution mass spectra were performed on an Agilent 6210 TOF LC/MS using ESI-MS. All anhydrous reactions described were performed under an atmosphere of argon using oven-dried glassware. Thin-layer chromatography (TLC) was performed on aluminum-backed TLC plates pre-coated with Merck silica gel 60 F254. Compounds were visualized with UV light and/or staining with phosphomolybdic acid (5% solution in EtOH). Normal phase column chromatography was carried out using Avanco or Merck silica gel 60 (230-400 mesh) or by Combiflash nextgen plus from Teledyne using preloaded silica gel cartridges 4g, 12g, 24g, and 40g from Teledyne. To concentrate by removal of solvents a Büchi rotary evaporator or a Büchi V-100 pump was used. All reagents and starting materials were analytical grade or better and were purchased from Sigma Aldrich, Alfa Aesar, TCI America, Combi-Blocks, or Arcos and used without further purification unless noted otherwise. All solvents were purchased from Sigma Aldrich, EMD, Anachemia, Caledon, Fisher or ACP and used without further purification unless otherwise specified. Tetrahydrofuran (THF) was freshly distilled over Na metal/benzophenone. Dichloromethane was dried and distilled over calcium hydride. Cold temperatures were maintained by use of the following conditions: 0 °C, ice-water bath; For reactions/work-up run-in water (H_2O), deionized water was used unless stated otherwise. Room temperature (rt) is defined as 23–25 °C. Abbreviations used as follows: – AcOH: acetic acid; $CHCl_3$: chloroform; DCM or CH_2Cl_2: dichloromethane; DMAP: 4-(Dimethylamino)pyridine; EtOAc or EA: ethyl acetate; MeOH: methanol or methyl alcohol; $NaHCO_3$: sodium bicarbonate; NaOMe: sodium methoxide; Na_2SO_4: sodium sulfate; THF: tetrahydrofuran; trityl chloride: Triphenylmethyl chloride; TMSOTf: trimethylsilyl trifluoromethanesulfonate.

6-O-trityl-1,2-dideoxy-2'-methyl-α-D-glucopyrano-[2,1-d]-Δ2'-thiazoline (**2**): To a stirred solution of GlcNAc-thiazoline (Knapp *et al.*, 1996) (0.500 g, 1 equiv., 2.28 mmol) in anhydrous pyridine (11.4 mL, 0.2 molar, 5mL/mmol) at 25 °C under argon atmosphere was added trityl chloride (763 mg, 1.2 equiv., 2.74 mmol) followed by DMAP (27.9 mg, 0.1 equiv., 228 μmol) at 25 °C. This reaction mixture was then stirred for 14h at 25 °C and the reaction progress was monitored by TLC. After completion of the reaction, the reaction mixture was poured on 50 mL of ice-cold water and then extracted with EtOAc (50 mL x 3). The organic extracts were combined, washed with water 50 mL x 2, brine 50 mL and then dried over anhydrous sodium sulfate. The volatiles were removed under reduced pressure to afford crude product that was kept under a high vacuum to remove traces of pyridine. The crude product was further purified on Combiflash by using 20% to 75% ethyl acetate in hexanes as a gradient to afford the desired compound **2** (0.75 g, 1.6 mmol, 71 %).

2: ^1H NMR (500 MHz, DMSO-d_6) δ 7.39 (d, J = 7.8 Hz, 6H), 7.33 (t, J = 7.5 Hz, 6H), 7.28 – 7.22 (m, 3H), 6.27 (d, J = 6.7 Hz, 1H), 5.26 (d, J = 4.5 Hz, 1H), 5.03 (d, J = 5.2 Hz, 1H), 4.19 (bs, 1H), 3.94 –

3.86 (m, 1H), 3.36 – 3.46 (m, 2H), 3.14 (t, *J* = 7.94, 7.20 Hz, 1H), 3.01 (d, *J* = 9.8 Hz, 1H), 2.21 (s, 3H).

HRMS (ESI$^+$) m/z calcd. For $C_{27}H_{28}NO_4S$ $(M+H)^+$ 462.1734, Found 462.1738; m/z calcd. For $C_{27}H_{27}NO_4SNa$ $(M+Na)^+$ 484.1553, Found 484.1552.

6-O-trityl-3,4-di-O-benzoyl-1,2-dideoxy-2'-methyl-α-D-glucopyrano-[2,1-d]-Δ2'-thiazoline (**3**) (Reynolds & Evans, 1942, Maiti *et al.*, 2007) : To a stirred solution of compound **2** (0.650 g, 1 equiv., 1.41 mmol) in anhydrous pyridine (7.04 mL, 0.2 molar, 5 mL/mmol) at 25 °C under argon atmosphere was added Benzoic anhydride (956 mg, 798 µL, 3 equiv., 4.22 mmol) followed by DMAP (17.2 mg, 0.1 equiv., 141 µmol) at 25 °C. The reaction mixture was then stirred for 14h at 25 °C and the reaction progress was monitored by TLC. After completion of the reaction, the reaction mixture was poured on 50 mL of ice-cold water and then extracted with EtOAc (50 mL x 3). The organic extracts were combined, washed with water 50 mL x 2, saturated NaHCO3 50 mL x 3, brine 50 mL, and then dried over anhydrous sodium sulfate. The volatiles were removed under reduced pressure to afford crude product that was kept under a high vacuum to remove traces of pyridine. The crude product was further purified on Combiflash by using 5% to 30% ethyl acetate in hexanes as a gradient to afford the desired compound **3** (0.59 g, 0.89 mmol, 63 %).

3: ^{1}H NMR (500 MHz, DMSO-d_6) δ 8.07 (d, *J* = 7.1 Hz, 2H), 7.85 (d, *J* = 7.1 Hz, 2H), 7.74 (t, *J* = 7.4 Hz, 1H), 7.69 (t, *J* = 7.4 Hz, 1H), 7.61 (t, *J* = 7.7 Hz, 2H), 7.54 (t, *J* = 7.8 Hz, 2H), 7.31 – 7.24 (m, 5H), 7.21 – 7.13 (m, 10H), 6.62 (d, *J* = 7.1 Hz, 1H), 5.71 (dd, *J* = 3.3, 1.8 Hz, 1H), 5.46 (d, *J* = 8.9 Hz, 1H), 4.78 – 4.72 (m, 1H), 3.64 (dt, *J* = 8.2, 3.7 Hz, 1H), 3.23 (dd, *J* = 10.8, 2.9 Hz, 1H), 3.16 (dd, *J* = 10.8, 4.5 Hz, 1H), 2.31 (d, *J* = 2.2 Hz, 3H).

HRMS (ESI$^+$) m/z calcd. For $C_{41}H_{36}NO_6S$ $(M+H)^+$ 670.2258, Found 670.2231; m/z calcd. For $C_{41}H_{35}NO_6SNa$ $(M+Na)^+$ 692.2077, Found 692.2050.

3,4-di-O-benzoyl-1,2-dideoxy-2'-methyl-α-D-glucopyrano-[2,1-d]-Δ2'-thiazoline (**4**): To a stirred solution of compound **3** (0.550 g, 1 equiv., 821 µmol) in an anhydrous DCM (8.21 mL, 0.1 molar, 10 mL/mmol) and methanol (4.11 mL, 0.2 molar, 5 mL/mmol) at 25 °C was added *p*-Toluenesulfonicacidmonohydrate (46.9 mg, 37.8 µL, 0.3 equiv., 246 µmol) under argon atmosphere. The reaction mixture was then stirred for 4h at 25 °C, and the reaction progress was monitored by TLC. After completion of the reaction, the reaction mixture was poured into 50 mL of ice-cold water and then extracted with DCM (50 mL x 3). The organic extracts were combined, washed with water 50 mL x 2, saturated NaHCO3 (50 mL x 2), brine 50 mL, and then dried over anhydrous sodium sulfate. The volatiles were removed under reduced pressure to afford crude product that was further purified on Combiflash by using 5% to 40% ethyl acetate in hexanes as a gradient to afford the desired compound **4** (0.20 g, 0.48 mmol, 58 %).

4: ^{1}H NMR (600 MHz, Acetone-d_6) δ 8.10 (dd, J = 8.3, 1.4 Hz, 2H), 8.05 (dd, J = 7.1, 1.1 Hz, 2H), 7.71 – 7.64 (m, 2H), 7.57 – 7.50 (m, 4H), 6.59 (d, J = 7.2 Hz, 1H), 5.92 (dd, J = 3.3, 1.7 Hz, 1H), 5.41 (dt, J = 8.2, 1.6 Hz, 1H), 4.80 (dtt, J = 5.8, 2.4, 1.2 Hz, 1H), 3.98 (t, J = 5.8 Hz, 1H), 3.78 – 3.66 (m, 3H), 2.35 (d, J = 2.3 Hz, 3H).

^{13}C NMR (151 MHz, Acetone-d_6) δ 167.25, 165.76, 165.51, 134.32, 134.30, 130.78, 130.71, 130.58 (2C), 130.50 (2C), 129.48 (2C), 129.47 (2C), 89.94, 77.78, 72.29, 71.61, 70.70, 63.15, 20.56.

HRMS (ESI^{+}) m/z calcd. For $C_{22}H_{22}NO_6S$ $(M+H)^+$ 428.1162, Found 428.1164; m/z calcd. For $C_{22}H_{21}NO_6SNa$ $(M+Na)^+$ 450.0982, Found 450.0977.

{(2-acetamido-2-deoxy-3,4,6-tri-O-acetyl-β-D-glucopyranosyl)}-(1→6)-3,4-di-O-benzoyl-1,2-dideoxy-2'-methyl-α-D-glucopyrano-[2,1-d]-Δ2'-thiazoline (**5**) (Jiang *et al.*, 2004) : To a stirred solution of compound **4** (0.100 g, 1 equiv., 234 μmol) in an anhydrous THF (2.34 mL, 0.1 molar, 10 mL/mmol) was added 2-acetamido-2-deoxy-3,4,6-tri-O-acetyl-α-D-glucopyranosyl-1-trichloroacetimidate (575 mg, 5 equiv., 1.17 mmol) in THF (2.34 mL, 0.1 molar, 10 mL/mmol) mixture at 25 °C under argon atmosphere. The reaction mixture was cooled to -20 °C and stirred for 30 minutes. Trimethylsilyl triflate (5.20 mg, 4.32 μL, 0.1 equiv., 23.4 μmol) was added to the reaction mixture at -20 °C. The reaction mixture was then stirred for 2h at -20 °C and the reaction progress was monitored by TLC. After 2h, the reaction mixture was poured on 20 mL of ice-cold water and then extracted with EtOAc 50 mL x 3. The organic extracts were combined, washed with water 50 mL x 2, saturated NaHCO3 50 mL x 3, brine 50 mL and then dried over anhydrous sodium sulfate. The volatiles were removed under reduced pressure to afford crude product that was used for the next reaction without further purification.

HRMS (ESI^{+}) m/z calcd. For $C_{36}H_{41}N_2O_{14}S$ $(M+H)^+$ 757.2273, Found 757.2264; m/z calcd. For $C_{36}H_{40}N_2O_{14}SNa$ $(M+Na)^+$ 779.2092, Found 779.2090; m/z calcd. For $C_{36}H_{40}N_2O_{14}SK$ $(M+K)^+$ 795.1832, Found 795.1824.

{(2-acetamido-2-deoxy-β-D-glucopyranosyl)}-(1→6)-1,2-dideoxy-2'-methyl-α-D-glucopyrano-[2,1-d]-Δ2'-thiazoline (**6**): To a stirred solution of compound **5** (0.035 g, 1 equiv., 46 μmol) in anhydrous methanol (4.6 mL, 0.01 molar, 100 mL/mmol) was added sodium methoxide (2.5 mg, 1 equiv., 46 μmol) at 25 °C under argon atmosphere. The reaction mixture was stirred at 25 °C for 16h. The reaction progress was monitored by TLC. After 16h, Amberlite IR-120 (H^+) ion exchange resin (100 mg) was added to the reaction mixture and stirred for 30 minutes. The resin was removed via filtration and the organic filtrate was concentrated under reduced pressure to afford crude product that was further purified on Combiflash by using 5% to 30% methanol in DCM as a gradient to afford the desired compound **6** (10.7 mg, 25.3 μmol, 55 %).

6: ^{1}H NMR (600 MHz, CD_3OD) δ 6.30 (d, J = 6.8 Hz, 1H), 4.43 (d, J = 8.4 Hz, 1H), 4.31 – 4.26 (m, 1H), 4.07 (t, J = 4.8 Hz, 1H), 4.01 (dd, J = 11.4, 2.2 Hz, 1H), 3.87 (dd, J = 11.9, 2.2 Hz, 1H), 3.71 –

3.62 (m, 3H), 3.60 (dd, J = 9.1, 4.6 Hz, 1H), 3.48 – 3.41 (m, 2H), 3.27 – 3.17 (m, 3H), 2.26 (d, J = 2.0 Hz, 3H), 2.02 (s, 3H).

^{13}C NMR (151 MHz, CD_3OD) δ 170.94, 167.41, 100.44, 87.18, 78.52, 75.10, 72.98, 72.83, 71.12, 69.20, 68.23, 67.99, 59.88, 54.47, 20.29, 17.55.

HRMS (ESI$^+$) m/z calcd. For $C_{16}H_{27}N_2O_9S$ $(M+H)^+$ 423.1432, Found 423.1441; m/z calcd. For $C_{16}H_{26}N_2O_9SNa$ $(M+Na)^+$ 445.1251, Found 445.1260.

S2. NMR spectra

S2.1. ^{1}H NMR of compound 6.

SBB-3-130-5_600_THIAZOLINE-DI-GLUCNAC.1.fid

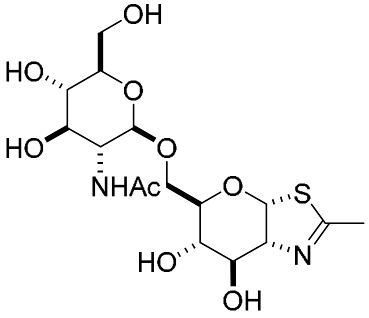

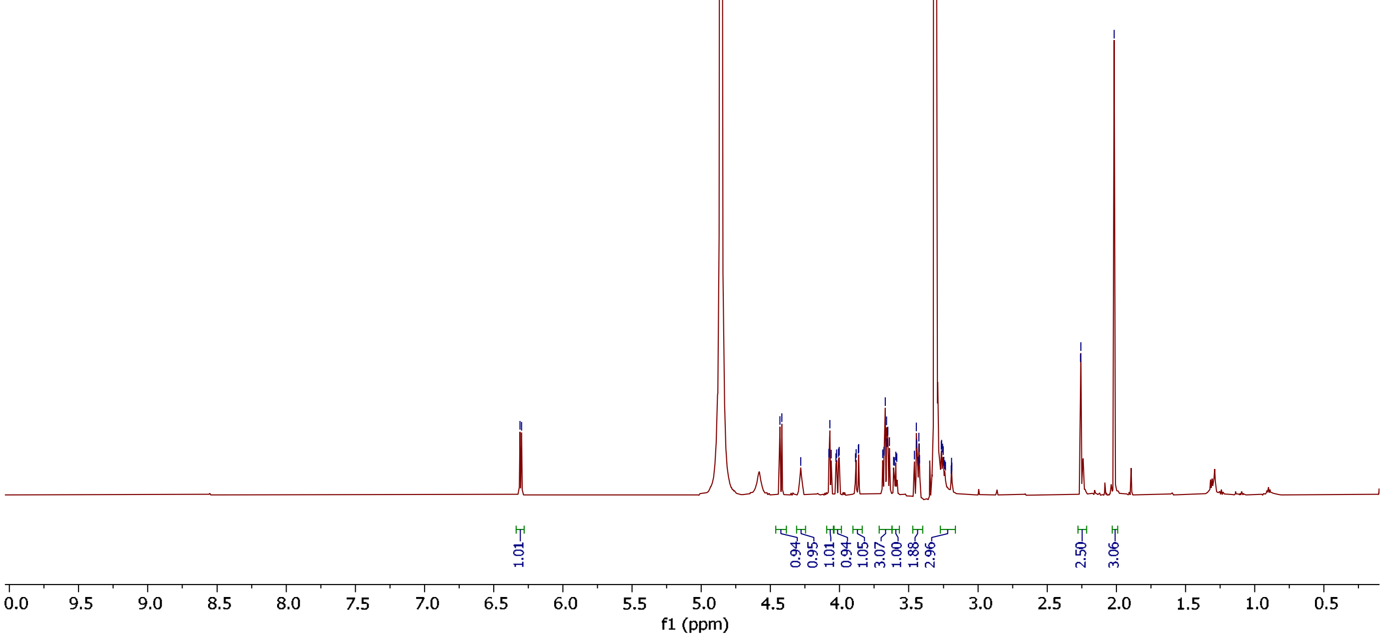

S2.2. ^{13}C NMR of compound 6

SBB-3-130-5_600_THIAZOLINE-DI-GLUCNAC.2.fid

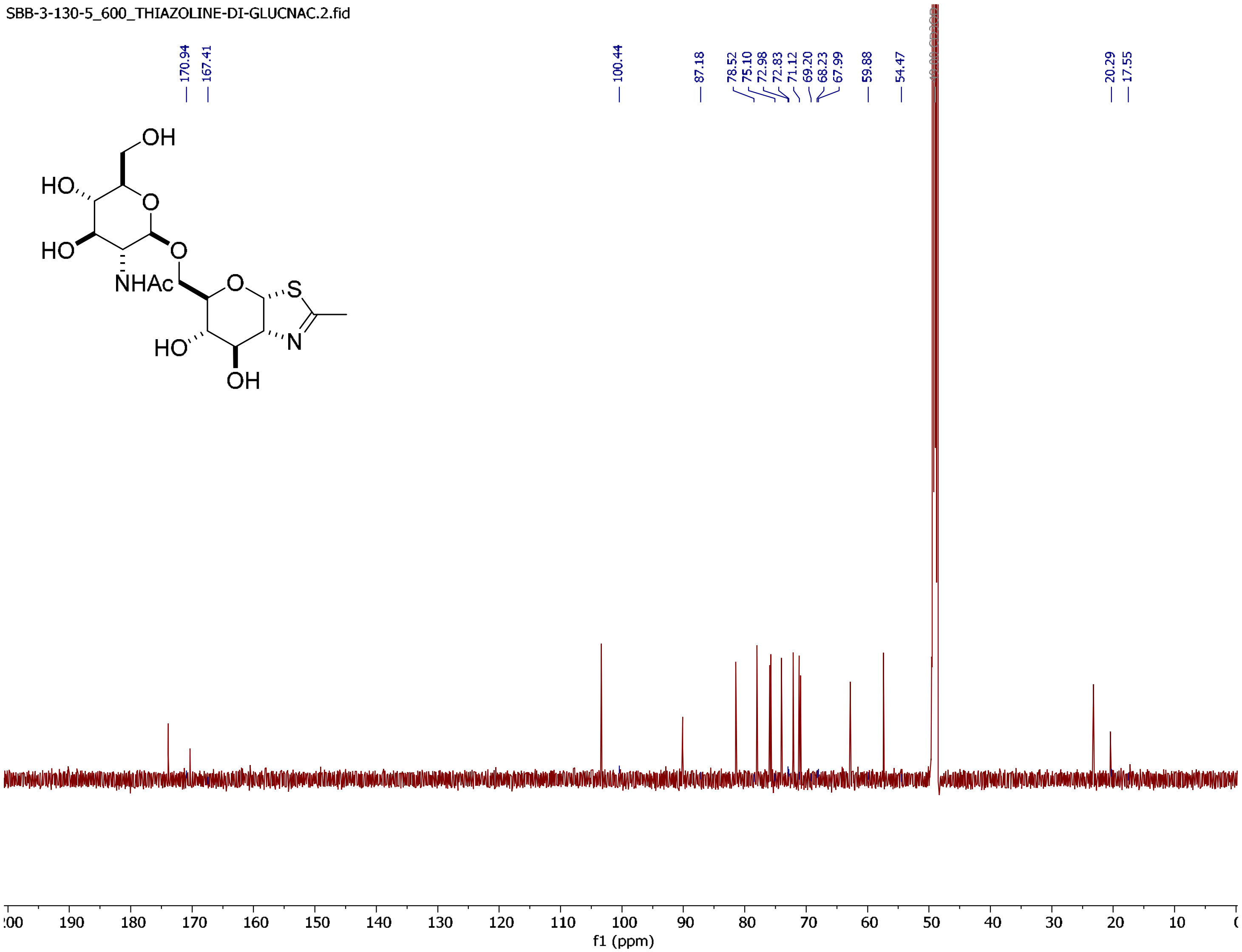

S2.3. ^{1}H NMR of compound 4

SBB-3-102_600.1.fid

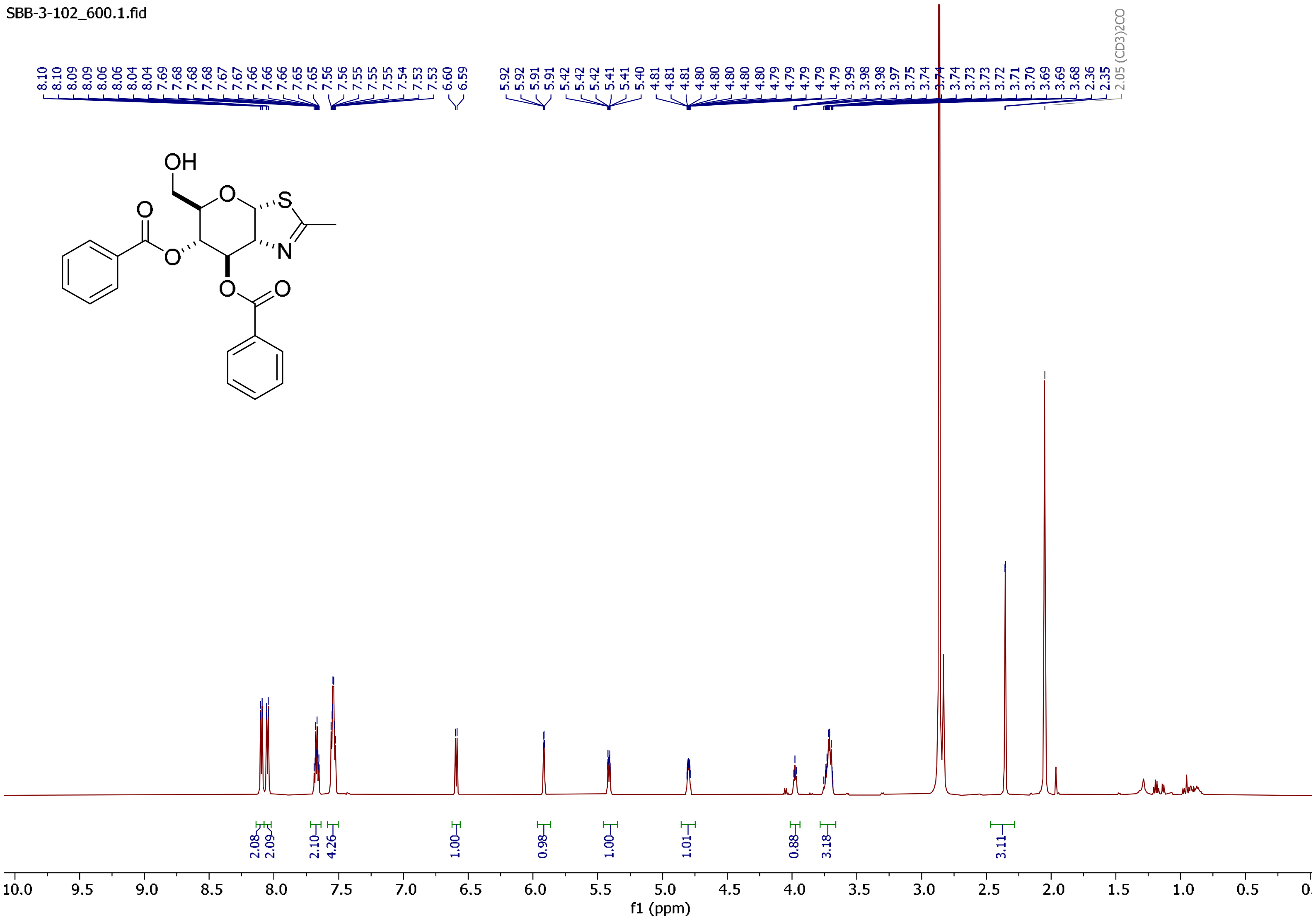

S2.4. ^{13}C NMR of compound 4

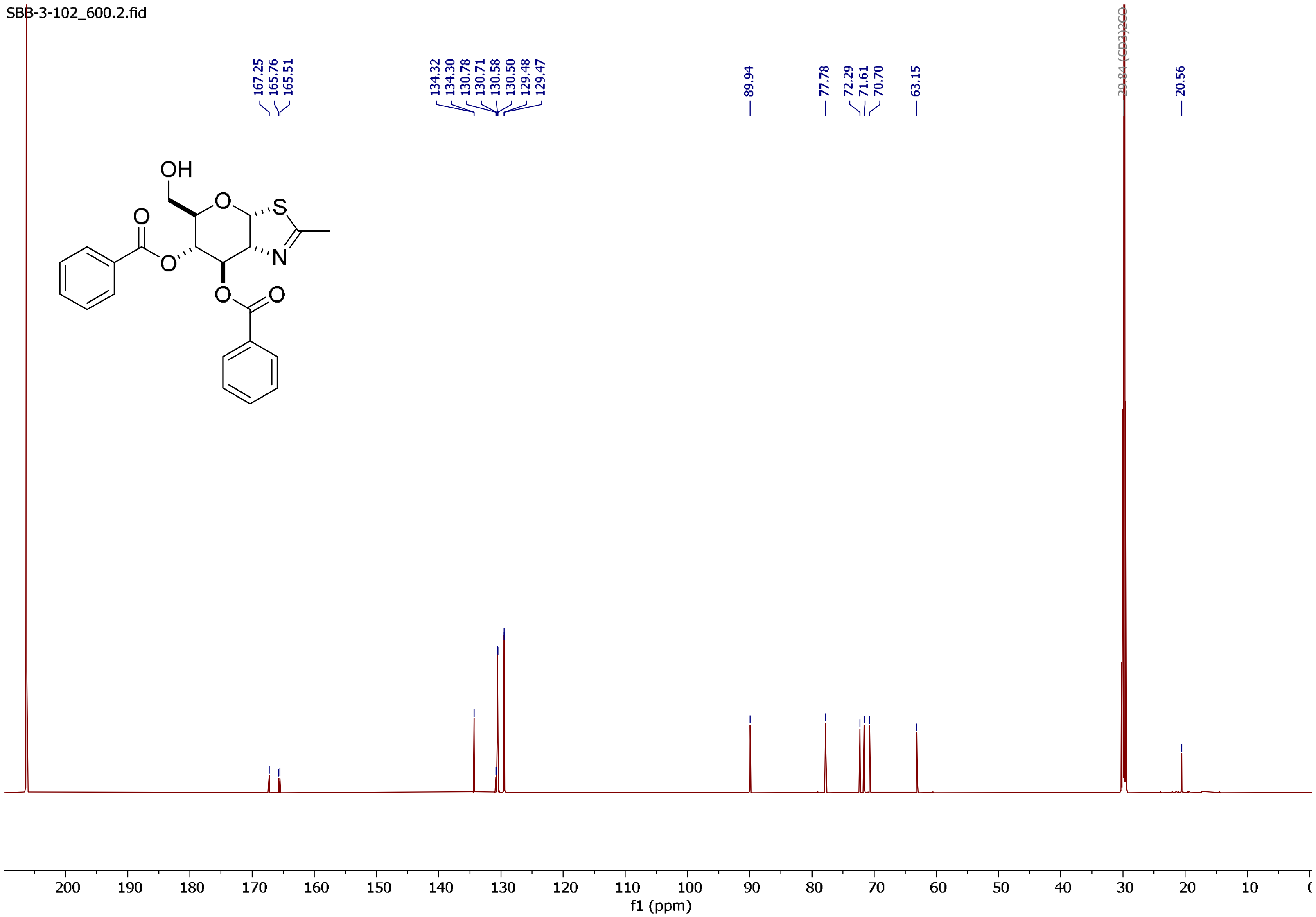

S2.5. ^{1}H NMR of compound 3

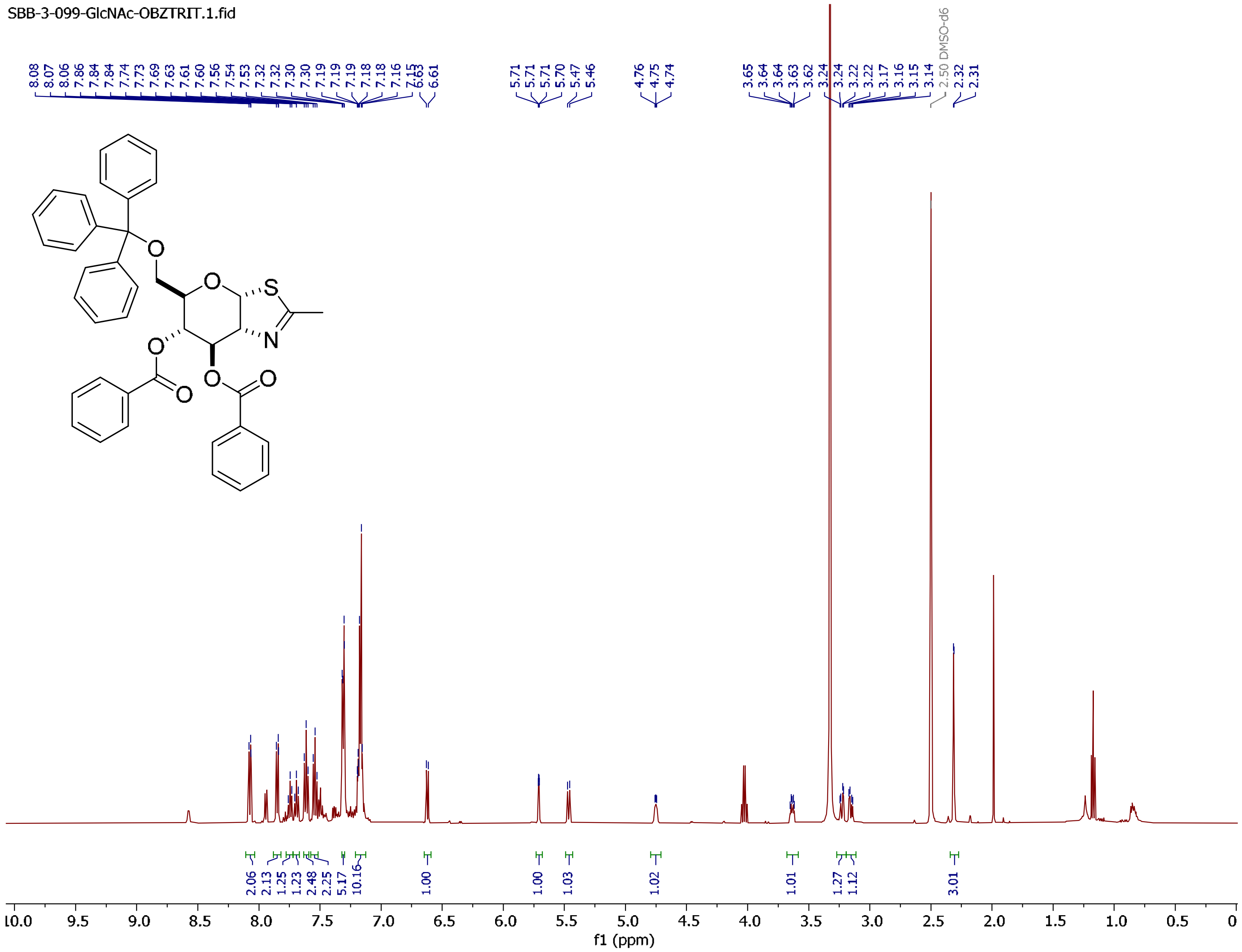

S2.6. ^{1}H NMR of compound 2

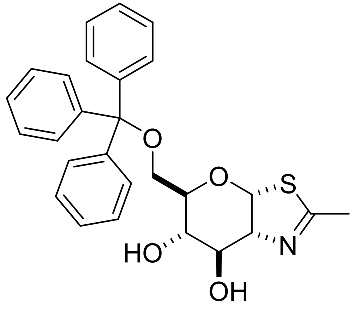

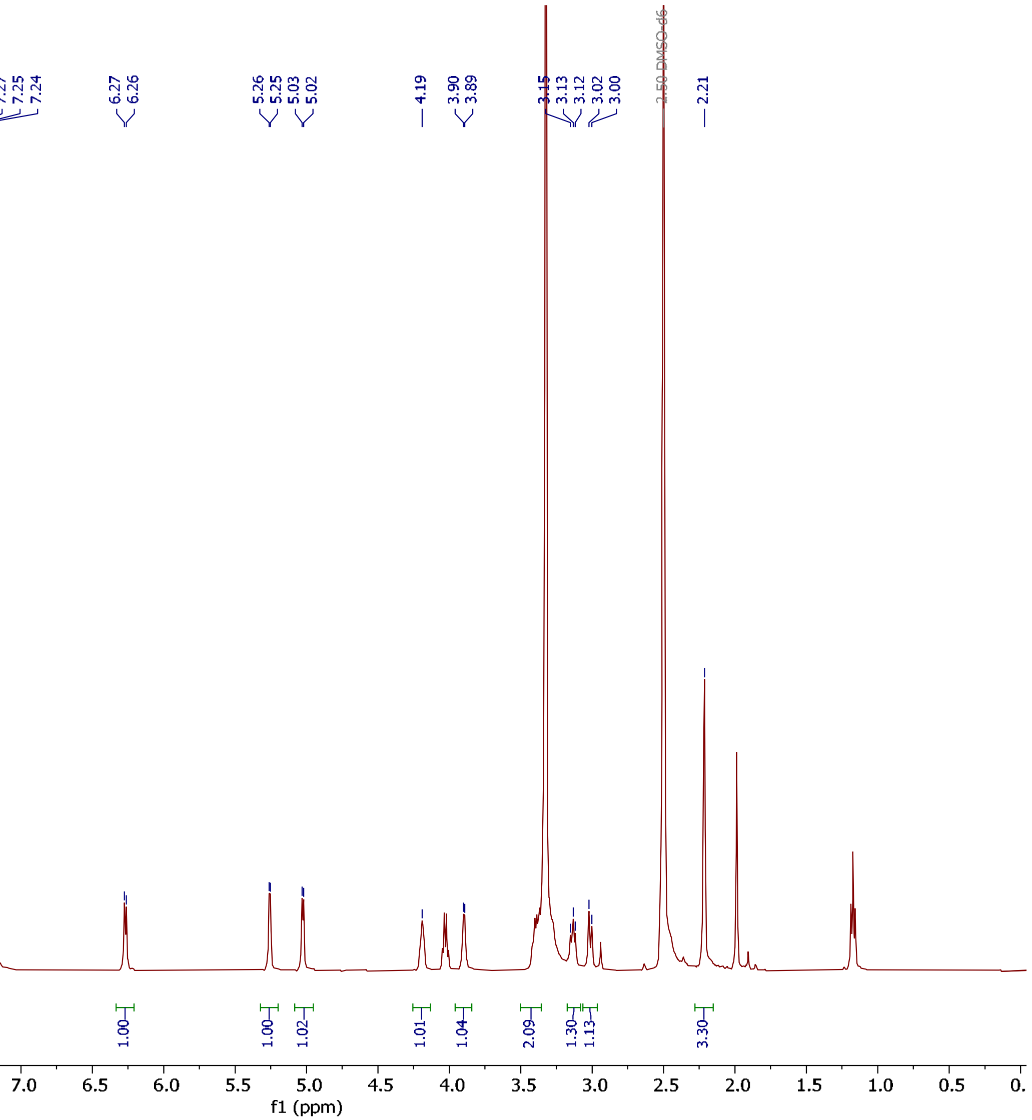

References

Fulmer, G. R., Miller, A. J. M., Sherden, N. H., Gottlieb, H. E., Nudelman, A., Stoltz, B. M., Bercaw, J. E. & Goldberg, K. I. (2010). *Organometallics* **29**, 2176-2179.

Jiang, Z.-H., Gandhi, S. & Koganty, R. R. (2004).

Knapp, S., Vocadlo, D., Gao, Z. N., Kirk, B., Lou, J. P. & Withers, S. G. (1996). *J Am Chem Soc* **118**, 6804-6805.

Krissinel, E. (2012). *J Mol Biochem* **1**, 76-85.

Maiti, K. K., Lee, W. S., Takeuchi, T., Watkins, C., Fretz, M., Kim, D. C., Futaki, S., Jones, A., Kim, K. T. & Chung, S. K. (2007). *Angew Chem Int Edit* **46**, 5880-5884.

Reynolds, D. D. & Evans, W. L. (1942). *Org Synth* **22**, 56-58.

Varadi, M., Bertoni, D., Magana, P., Paramval, U., Pidruchna, I., Radhakrishnan, M., Tsenkov, M., Nair, S., Mirdita, M., Yeo, J., Kovalevskiy, O., Tunyasuvunakool, K., Laydon, A., Zídek, A., Tomlinson, H., Hariharan, D., Abrahamson, J., Green, T., Jumper, J., Birney, E., Steinegger, M., Hassabis, D. & Velankar, S. (2023). *Nucleic Acids Research*.
